# Enhancing *De Novo* Designed Peptides and Proteins *via* Irreversible Covalent Isoquinolinium Capture

**DOI:** 10.1021/acschembio.6c00584

**Published:** 2026-09-18

**Authors:** Paul M. Levine, Patrick W. Erickson, Timothy W. Craven, Aaron T. Balana, Derrick R. Hicks, Green Ahn, Chan J. Kim, Lisa Brandenburg, Wei Yang, Danielle P. Johnson Erickson, David Baker

**Affiliations:** 1 Department of Biochemistry and Institute for Protein Design, University of Washington, Seattle, Washington 98195, United States; 2 HHMI, University of Washington, Seattle, Washington 98195, United States; 3 Department of Radiation Oncology, School of Medicine, University of Washington, Seattle, WA 98195, USA

## Abstract

Irreversible covalent inhibitors have garnered significant attention in recent years. Despite encouraging progress, the vast majority contain electrophiles that target the least abundant amino acid cysteine, substantially limiting target inhibitor design for therapeutic intervention. Here, we generalize 2-ethynylbenzaldehyde as a proximity-induced electrophile for generating irreversible covalent peptide and protein inhibitors that specifically target native lysine residues. Leveraging this warhead, we design a covalent *de novo* peptide that potently engages MCL1 to block its interaction with Bak. We show it is faster, more selective, and increases potency 61-fold for MCL1 relative to a sulfonyl fluoride warhead. Additionally, with the guide of a computational script to predict ‘reactive hotspots’ at the protein level, we develop a minibinder that labels PD-L1 *in vitro* and in live cells, displays a slower off-rate, and potently blocks the native PD-1 and PD-L1. These results establish isoquinolinium capture as a promising strategy to inhibit protein-protein interactions and for the development of novel covalent peptide and protein therapeutics.

## Graphical Abstract

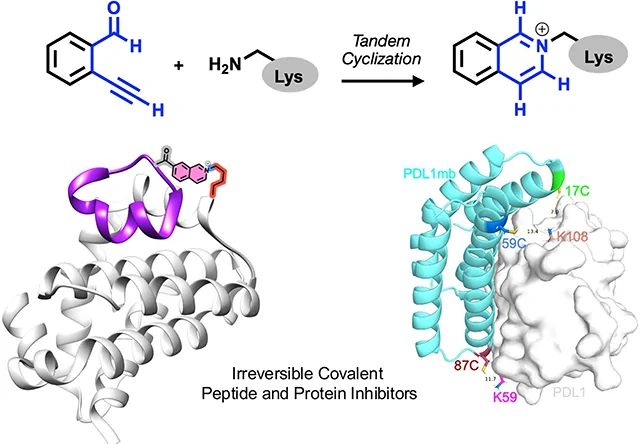

## Introduction

Cyclic peptides and miniproteins are becoming increasingly attractive for drug discovery as they have the capacity to modulate complex protein–protein interactions with remarkable potency and selectivity.^1^ Their size and tunability present opportunities to engineer stability and other favorable pharmacological characteristics, however, their inherent short circulation times can often make this challenging.^2^ While some methods can overcome stability, such as introduction of non-canonical amino acids or half-life extenders, there isn’t a predictable strategy to make these substitutions.^3^ One approach gaining traction to evade rapid renal clearance is by exploiting proximity-induced latent reactivity to generate covalent drugs.^4,5^ Covalent mechanisms largely circumvent short half-lives *in vivo* as they decouple drug efficacy from pharmacokinetics, resulting in prolonged target engagement and enhance drug binding off-rates.^6^ While significant progress has been made, the vast majority of these chemistries, such as acrylamides, acetamides, and vinyl sulfonamides, target cysteine.^7–9^ This substantially limits covalent inhibitor design for many potential targets as cysteine is the least abundant amino acid (~1.9% in proteins) and is often not found at protein-protein interfaces or active sites outside of cysteine proteases.^10^

As the field continues to mature, extensive research is being focused on preparing a variety of electrophiles that can be introduced chemically or genetically for targeting other nucleophilic amino acids more commonly found in proteins, such as lysine, tyrosine, and histidine.^11^ The most common method used to target these residues is sulfur (VI) fluoride exchange (SuFEx) chemistry, which was recently used by the Pentelute lab to inhibit binding between CD94-NKGA and HLA-E *via* chemical installation of a sulfonyl fluoride warhead.^12^ Moreover, the Wang lab has pioneered the genetic incorporation of aryl fluorosulfonate warheads to inhibit the interaction between PD-L1 and PD-1.^13^ Although promising, multiple factors must be considered when harnessing SuFEx electrophiles, including sulfonyl fluoride susceptibility to hydrolysis, low intrinsic reactivity of aryl fluorosulfonates often enhanced by the requirement of a neighboring local basic arginine residue, and potential proteome-wide promiscuity.^14,15^

More recently, 2-acetyl phenylboronic acid and ethyl 2-(bromomethyl)acrylate electrophilic warheads were introduced to target lysine, however 2-acetyl phenylboronic binds reversibly which is likely to display diminished pharmacokinetic profiles *in vivo* and ethyl 2-(bromomethyl)acrylate has cross-reactivity with cysteine.^16,17^ In general, many lysine specific methods exist, however most either generate highly heterogeneous products, are reversible, and have not been adopted beyond small molecules.^18–20^ In contrast, 2-ethynylbenzaldehyde (EBA) was recently shown to selectively and irreversibly modify primary amines (mainly N-terminal) *via* isoquinolinium formation with high efficiency at physiological pH.^21^ In a subsequent report, EBA was introduced into various small molecule activity based probes to engage internal lysine residues and profile the kinome to generate inhibitors of two kinases, tyrosine-protein kinase (ABL1) and epidermal growth factor receptor (EGFR).^22^ Despite potential metabolic issues, aldehydes have been active pharmaceutical ingredients.^23^

Building on this foundation with very limited small molecule examples in literature, which require lengthy chemical transformations and do not contain chemical handles for incorporation of EBA into peptide or proteins, here we expand the scope of irreversible lysine chemistry to rapidly introduce this warhead into biomolecules under biocompatible aqueous conditions for proximity-induced covalent capture. In one example, we apply this warhead to generate irreversible covalent inhibitors against hMcl-1, a key protein that prevents apoptosis and is often overexpressed in various cancers.^24^ We demonstrate, relative to a sulfonyl fluoride warhead, that the reaction produces greater protein stabilization and potently blocks its interaction with Bak. In a separate example, with the guide of a computational script for identifying ‘reactive hotspots’, we generate a potent and covalent miniprotein binder that engages PD-L1 to block its interaction with PD-1.

## Results

We previously introduced a denoising diffusion-based method for accurate *de novo* cyclic peptide design, dubbed RFpeptides, and identified cyclic peptide inhibitors that bound to the MCL1–BH3 interaction site.^25^ The best design for this target, MCB_D2 (cyclo[PPEIAWLADAVGLKDA]) displayed a binding affinity of 2 μM *via* surface plasmon resonance (SPR). Moreover, upon co-crystallization, we noticed ‘hotspot’ K234 in MCL1, which has previously been established as a nucleophilic capture site, was in very close proximity (~8.1 Å) to a solvent exposed lysine in MCB_D2 (SI Fig. 1). We hypothesized that outfitting this lysine in the peptide, or shorter Dap or Dab derivatives, with an EBA electrophilic warhead could irreversibly capture K234 in MCL1 and potently block its interaction with endogenous substrates for potential anticancer therapy development.

For our approach to modify peptides with an EBA warhead, we envisioned generating reagents compatible with both on resin solid-phase peptide synthesis and in solution chemistry (Fig. 1A). For on resin chemistry, we synthesized **1** from 2-bromo-5-methylacetate benzaldehyde using trimethylsilyl acetylene *via* a Sonogashira cross coupling reaction. Following this, the trimethylsilyl and methyl ester protecting groups were removed with lithium hydroxide, providing the carboxylic acid for amide coupling. This can be used in combination with orthogonal amine protecting groups, such as ivDde or alloc, that can be selectively deprotected following synthesis for on resin amide condensation reactions. Additionally, incubation of **1** with *N*-hydroxysuccinimide and activator yielded reagent **2** for in solution installation *via* NHS chemistry.

With these warheads in hand, we chose to make 3 variants of MCB_D2 where the lysine either stays the same or is slightly shorter with a Dap or Dab substitution, as the co-crystal structure of MCB_D2 in complex with MCL1 is in very close proximity to K234 (~8.1 Å) and we wanted to investigate the effect of linker length. Accordingly, following Fmoc-based solid phase peptide synthesis and in solution cyclization, reagent **2** was incubated with the peptides in aqueous buffer, generating peptides **W1-W3** (Fig. 1B). We then purified each peptide by RP-HPLC and confirmed their identities by mass spectrometry (SI Fig. 2). Similarly, we synthesized sulfonyl fluoride variants of the same peptides to directly compare labeling between SuFEx and EBA. These peptides were amide coupled to 5-(fluorosulfonyl)-2-methoxybenzoic acid *via* selective deprotection of ivDde, generating peptides **W4-W6** (Fig. 1B & SI Fig. 3). Each peptide was then purified and characterized (SI Fig. 2) and **W1-W6** were subsequently evaluated for covalent protein modification (Fig. 1C).

Following peptide purification, cross-linking experiments were performed with **W1-W6** (20 μM, 10 equiv) and MCL1 (2 μM, 1 equiv) under physiologically relevant conditions in HEPES buffer (pH 7.4) at 37 °C. In all cases, EBA modified peptides **W1-W3** labeled MCL1 with increasing conversion as linker length increased, with **W3** showing the highest conversion at 2 h (Fig. 2). It is important to note that only a very small amount of doubly labeled protein was detected, suggesting EBA is a latent warhead and **W1-W3** have a very specific binding mode. Additionally, there was also a small adduct with a slightly higher molecular weight observed for EBA (+195 species with a molecular weight of 46, 563) that we were unable to identify, however it was quite minor. In contrast, SuFEx modified peptides **W4-W6** all completely modified MCL1 with varying degrees of singly and significantly higher doubly labeled protein. Labeling was also conducted at 1:1 and 1:5 equivalence under the same conditions as above (SI Fig. 4). Interestingly, EBA peptide **W3** labeled quicker than SuFEx peptide **W6** and only a very tiny amount of double labeling was observed at 5 equivalence. This data indicates that **W6** reacts slower than **W3** and at higher concentrations could potentially prove to be problematic in proteome-wide experiments as double labeling is more prominent with **W6** at 10 equivalence relative to **W3**. Moreover, to determine if K234 is the site of modification, we modified MCL-1 with a known substrate that covalently captures K234 (SI Fig. 5).^24^ Upon purification of pre-blocked K234, no additional labeling was found even with a 5-fold excess of **W3**, suggesting K234 is the intended site of modification.

To gain a better understanding of the biophysical properties of these **W3** and **W6** who share the same side-chain length, we employed a thermal shift assay to determine the temperature at which 50% of the protein is unfolded in the presence or absence of peptide (△*T*_*m*_). In this assay, 2 μM of the MCL1 protein is gradually heated in the presence of the peptides at indicated concentrations, as well as a fluorescent dye that binds hydrophobic regions that get exposed upon thermal melting. Protein unfolding is then monitored by an increase in fluorescence. At the highest concentration of peptide, MCB_D2, exhibited a △*T*_*m*_ = 2.8°C. The SuFEx peptide **W6** was comparable, eliciting a △*T*_*m*_ = 2.5°C (Fig. 3A). Surprisingly, EBA peptide **W3** generated a △*T*_*m*_ = 8°C relative to MCB_D2 and **W6**. It is possible that the elevated temperature used in this assay may have resulted in artifactual, nonspecific labeling of MCL1 that resulted in the drastic melting profile differences between the SuFEx and EBA-containing peptides. We speculate that the EBA warhead may have resulted in a larger extent of nonspecific modification, potentially explaining the stronger thermal stabilization. However, we believe that this effect is irrelevant in the intended use case (i.e. physiological temperatures) for this technology.

We then conducted SPR to measure the binding affinity of peptides **W3** and **W6** to MCL1 and evaluate the off-rates of the covalent binders relative to non-covalent MCB_D2 (Fig. 3B & SI Fig. 6). In contrast to the thermal shift experiment, we did not observe any indication of multiple labeling, since the highest concentrations of peptides used resulted in sensor responses that are within the theoretical maximum for 1:1 binding. Examination of measured on- and off-rates *via* NHS capture indicated that affinity differences are largely driven by improvements in the off-rate, consistent with covalent labeling. The EBA warhead imparts a ~2 orders of magnitude improvement while the SuFEx ~4 orders of magnitude. It must be noted that the dissociation of **W3** appears to deviate from the standard 1:1 fit and could potentially be multi-phasic. Possible explanations for this are (1) some of the bound peptide may not have fully reacted by the start of the dissociation phase due to slower reactivity or some amount of quenching, or (2) some covalent unstable intermediates (e.g. imines) slowly decompose and dissociate over time. The poor fit potentially may underestimate the measured k_off_’s and KD for **W3**. Meanwhile, **W6** exhibited dissociation rates that are too slow to be measured accurately by the instrument, hence its measured k_off_ and K_d_ may also be underestimated. Notably, determination of the half-maximal binding EC_50_ via steady-state equilibrium fitting of SPR response gives K_d_’s similar to the parent K_d_, suggesting that the inherent, non-covalent contacts between MCL1 and the peptides are essentially similar. Similarly, biotin capture produced a very comparable multi-phasic fit with an analogous off-rate (SI Fig. 7).

Endogenous binding of BH3 proteins, such as the Bak substrate, promotes apoptosis by interacting with and neutralizing anti-apoptotic MCL1. Strategies to mimic native BH3 function are therefore of great interest for potential cancer therapies. To assess the ability of **W1-W6** to bind and antagonize MCL1, a competitive homogeneous time resolved fluorescence (HTRF) binding assay was used. In this assay, competitive binding was detected by a change in fluorescence resonance energy transfer (FRET) that occurs upon displacement of a fluorescently labeled Bak substrate (K_d_ = ~200 nM) from terbium labeled MCL1. Relative to MCB_D2, which exhibited an IC_50_ = 8.9 μM, SuFEx peptide **W6** showed a 2.8 fold-increase in potency with an IC_50_ = 3.2 μM (Fig. 3D). Interestingly, EBA peptide **W3** elicited a 61 fold-increase and 22 fold-increase in potency (IC_50_ = 146 nM) relative to MCB_D2 and **W6**, respectively. This significant enhancement in activity for **W3** may be explained by its robust melting temperature which seems to stabilize MCL1 over SuFEx, likely due to local conformational dynamics upon protein binding. Moreover, this has been observed previously with a small molecule example.^22^

The scope of this technology reaches beyond synthetic peptides, such as through the generation of covalent miniproteins for therapeutic applications. To address this, we investigated a minibinder model system where we previously designed binders against the major oncogenic target programmed death-ligand 1 (PD-L1) to block its interaction between programmed cell death protein 1 (PD-1).^26^ To streamline this approach at the protein level, we created a new computational script termed ‘reactivity sphere’ which allows us to predict sites likely amenable for covalent modification based on distance, predicted monomer stability, and predicted binding ddG (Fig. 4A & SI Fig. 8). Specifically, the script takes a target/binder pdb complex and a list of target nucleophile types (i.e. Cys, Lys, His) to identify potential crosslinking sites where the Cb to Cb distance between the binder residue and target nucleophile is less than 11.5 Å and the Ca-Cb vector between the binder residue and target residue point towards each other. For each binder residue that satisfies these geometric requirements, we mutate the residue to Ala and Lys, repack the side-chain, and calculate the mutations impact on the binder monomer energy and binding energy. If either mutation disrupts the monomeric energy or binding energy by greater than 5 energy units, based on the rosetta energy function, we discard the residue. After the energy based filtering, a list of the remaining binder residues is output as potential sites for warhead incorporation.

Upon applying our reactivity sphere script, two lysine residues in PD-L1 passed our script threshold (K59 and K108) with 3 cysteine point mutants (Fig. 4B). As K17C and R87C were closer to loop regions and likely more flexible, we decided to mutate R59C to make MB1 targeting K108 as it was identified as a ‘reactive hotspot’ (Fig. 4B). In this system we are modifying an engineered cysteine point mutant and thus needed to generate a new heterobifunctional cross-linker for covalent capture. Starting from probe **1**, maleimide ethylamine was introduced into the EBA precursor under standard amide forming conditions with HATU, yielding probe **3** (Fig. 4C). With our design and probe in hand, we expressed, purified, and chemically installed probe **3** onto MB1 *via* malemide chemistry in aqueous buffer, generating MB1_EBA (SI Fig. 9).

Following protein purification to remove any excess probe, cross-linking experiments were performed by incubating human PD-L1 at 37°C with MB1_EBA at a 1:10 molar ratio to determine qualitative crosslinking efficiency by a time course gel electrophoresis assay (Fig. 4D). Under these conditions, we saw a new band correlating to covalent product at approximately 45–50 kDa. Since the minibinder has a tendency to form dimers in solution, some of the EBA reacted to form covalently linked minibinder dimers (~30 kDa). However, PD-L1 (~35 kDa with smearing observed from PTM's) was consumed to form a covalent complex with MB1_EBA. To further verify this, SPR was conducted and we observed the off-rate for MB1_EBA was significantly reduced relative to its cysteine mutant counterpart MB1, with basically no dissociation from PD-L1 as more complex remains bound after the same dissociation time (Fig. 4E & SI Fig. 10). To evaluate the ability of MB1_EBA to crosslink PD-L1 in live cells, a human melanoma cell line (A375 cells) expressing the protein was used. Analysis by western blot using an anti-PD-L1 antibody confirms some crosslinking occurs in cells expressing PD-L1 (Fig. 4F). To address selectivity, we generated a knock-out cell line from PD-L1 expressing MDA-MB-231 TNBC cells. Relative to PD-L1 expressing cells, which see a significant increase in fluorescence labeling upon anti-his-488 detection from minibinder labeling, we observed very minimal background labeling in knock-out cells which strongly indicates MB1_EBA is targeting PD-L1 as designed. We also incubated MB1_EBA with a control protein (SIRT5) containing multiple surface exposed lysine residues and observed no labeling (SI Fig. 11). Lastly, MB1_EBA potently blocks the interaction between PD-1 and PD-L1 *ex vivo* using a cell based luminescent assay. Relative to MB1, which produced an EC_50_ = 28.4 nM, MB1_EBA a comparable EC_50_ = 39.2 nM (Fig. 4G). Although there isn’t a significant increase in potency for PD-L1, which is often observed for covalent therapeutics in general, a reduced off-rate can still render desirable pharmacological characteristics.^27^ Taken together, these data strongly suggest that the warhead and computational script can be used to generate potent covalent inhibitors that disrupt the interaction between PD-1 and PD-L1.

In conclusion, we introduce EBA heterobifunctional electrophiles that can either be incorporated into peptides and proteins under aqueous biocompatible conditions for proximity-induced covalent capture. Expanding covalent lysine specific chemistry beyond small molecules is often hampered by difficulties associated with competing nucleophiles present in biomolecules and has also been limited to reversible covalent warheads. Harnessing these new warheads, we directly compare a peptide to a sulfonyl fluoride warhead for covalent modification of MCL1. Relative to SuFEx, we show EBA produces greater protein stabilization which results in a 61-fold improvement in activity. In a separate example, a computational script for identifying ‘reactive hotspots’ on proteins was developed to guide the design of PD-L1 covalent inhibitors. We show that labeling is efficient *in vitro*, specific in live cells relative to a PD-L1 knockout, displays a reduced off-rate, and still potently blocks the native PD-1 and PD-L1. EBA was also recently analyzed in a proteomic experiment which exhibited high lysine selectivity.^28^ While we and others show isolated instances of selectivity, it will be important to conduct a proteome wide selectivity screen for any assets moving toward clinical development. Taken together, these results strongly convey the utility of EBA chemistry for the design of irreversible covalent modulators that target lysine specifically and may be helpful where other lysine covalent chemistries may not be ideal. Moreover, we believe covalent chemistry is strongly dependent on many factors, such as choice of electrophile, linker length, and local environment. When employing these chemistries, it is important to consider the pros and cons for each. It is also unclear if peptides or proteins containing proximal lysine residues near the site of incorporation will self-react, this will likely be conformation dependent. In the future, we aim to broadly apply this technology to create various covalent peptide and protein therapeutics.

## Supplementary Material

Supplementary Material

**Supporting Information:** The Supporting Information is available free of charge at https://pubs.acs.org. Materials and methods, supplementary figures, and 1H NMR spectra for all compounds.

## Figures and Tables

**Figure 1.**
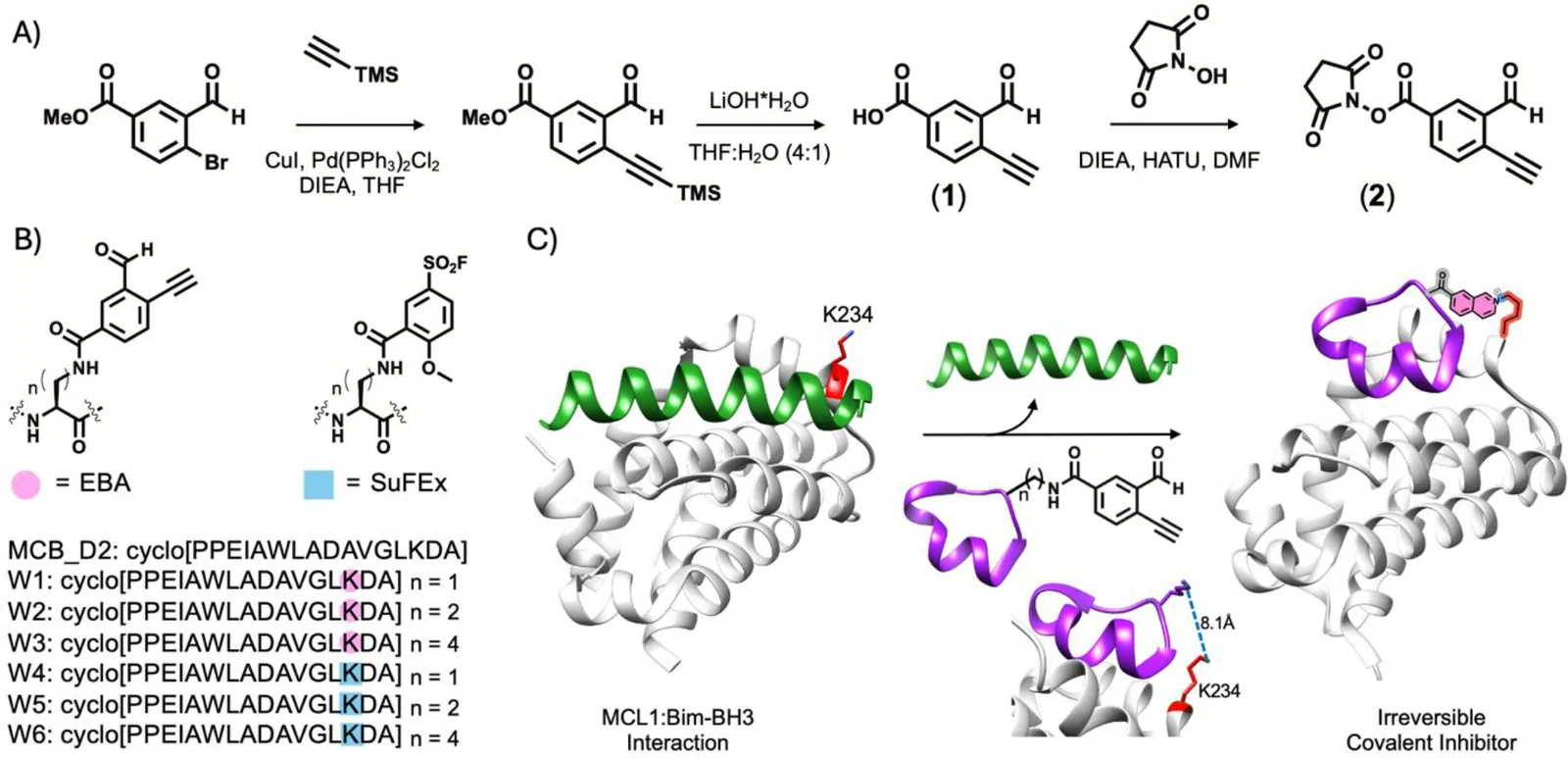
Design and synthesis of irreversible covalent peptides targeting MCL1. A) Synthesis of EBA warheads from 2-bromo-5-methylacetate benzaldehyde. B) Schematic of the covalent MCL1 peptides **W1**-**W6**, synthesized here, which contain either an EBA or SuFEx modification at the highlighted lysine (pink circles or blue squares). C) Schematic representation of cyclic peptides designed to covalently modify K234 in MCL1.

**Figure 2.**
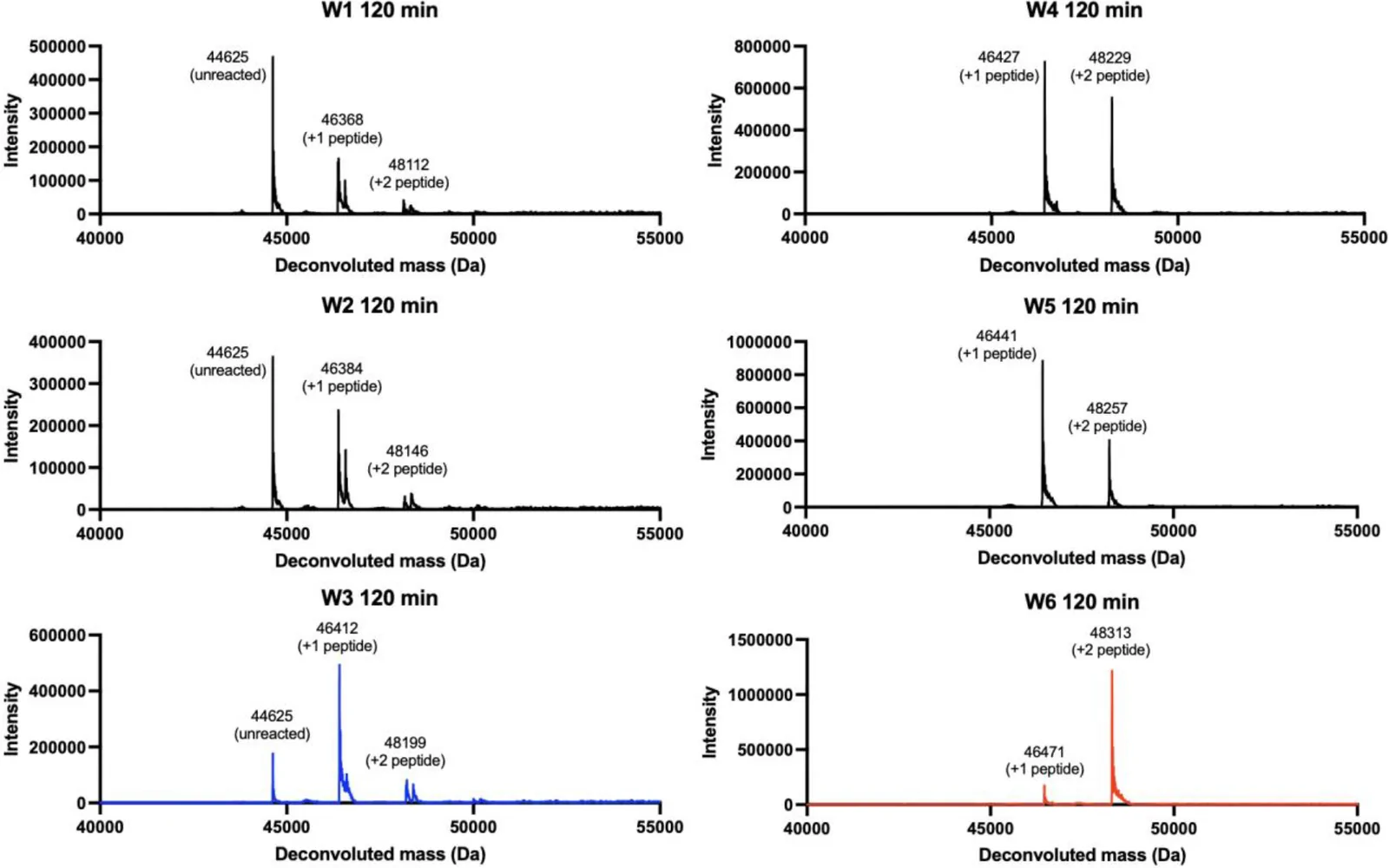
Peptide designs irreversibly crosslink MCL1. Cross-linking is evaluated with a 10-fold excess of the peptide (20 μM) over the target protein (2 μM) at physiological pH and temperature for 2 h. Deconvoluted mass spectra show highest conversion for singly labeled MCL1 is **W3** for EBA and **W5** for SuFEx, however in all cases SuFEx peptides **W4**-**W6** produce significantly more double labeled protein.

**Figure 3.**
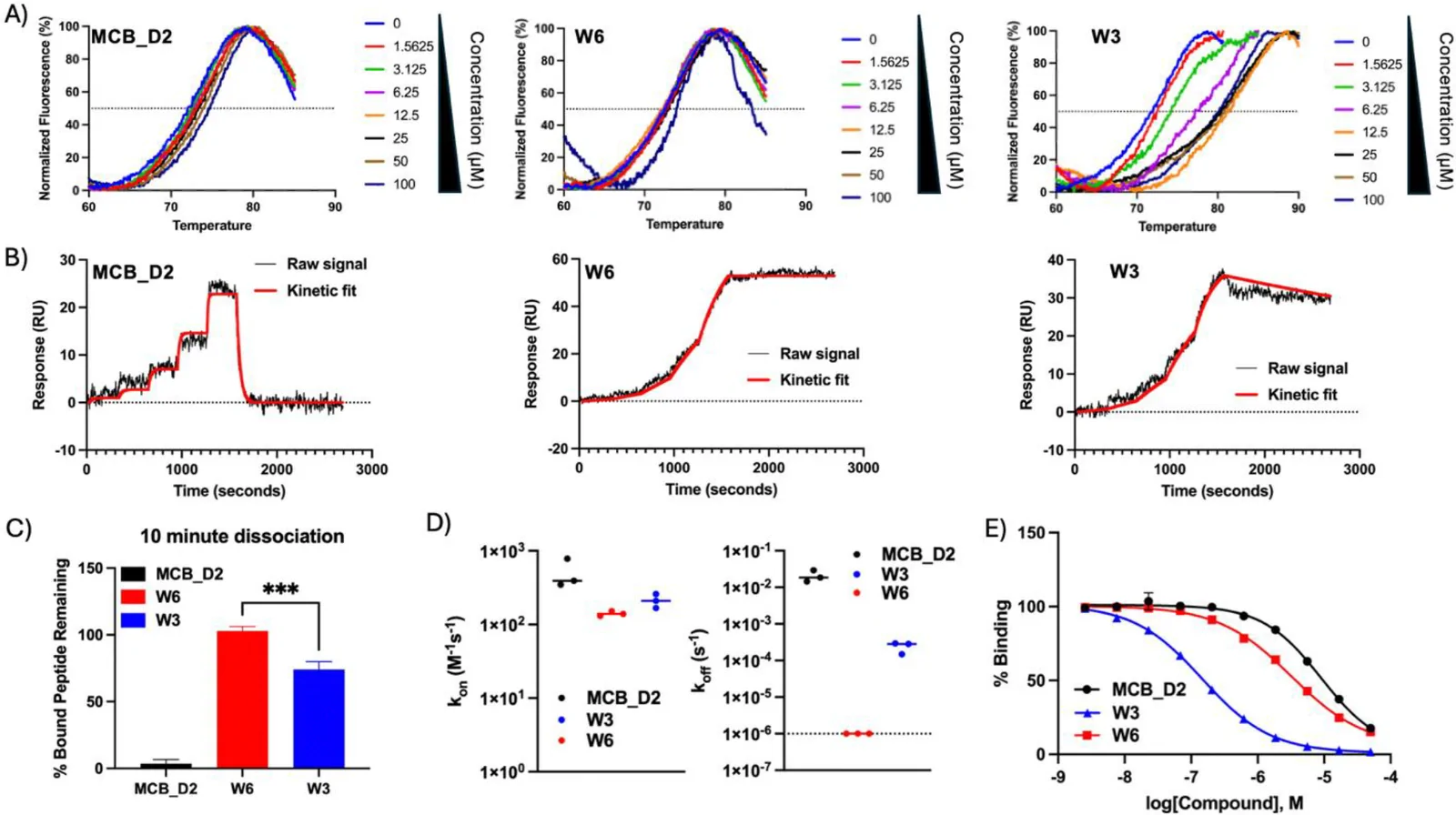
Biophysical and biochemical characterization of **W3** and **W6** on MCL1. A) Thermal stability of MCL in the presence of WT, **W3**, or **W6**. Relative to WT and **W6**, **W3** strongly stabilizes MCL1 with an 8°C shift in stability. B) Surface plasmon resonance (SPR) sensograms for indicated peptides. C) and D) SPR binding analysis showing complete reduction for non-covalent peptide bound to MCL1 following 10-minute dissociation, with almost no dissociation for **W6** and ~30% for **W3**. E) HTRF assay confirms **W3,** relative to MCB_D2 or **W6,** potently blocks the native interaction between a Bak peptide and MCL1. Data was collected in triplicate and data bars represent ± s.e.m.

**Figure 4.**
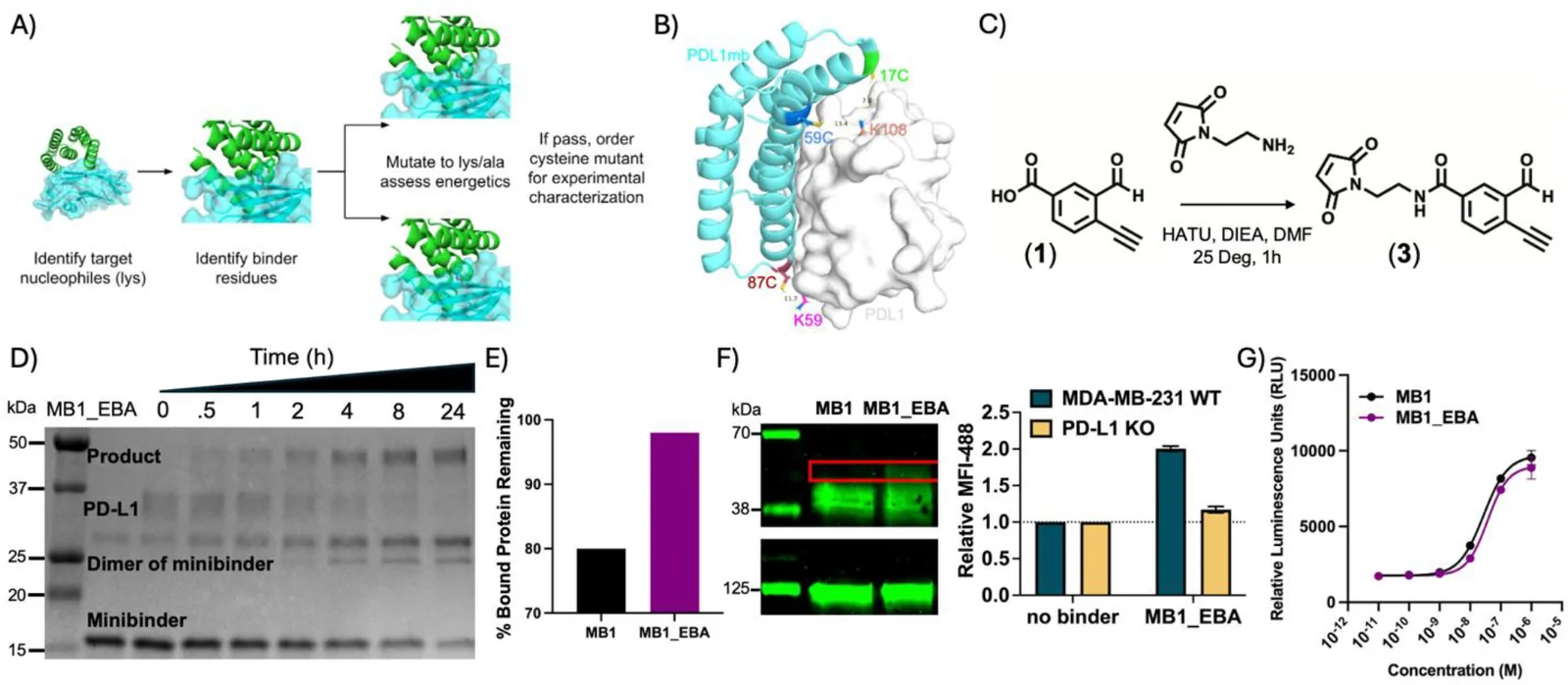
Computational ‘reactivity sphere’ script to predict potential hot-spot nucleophiles for covalent targeting and characterization of a designed covalent minibinder targeting PD-L1. A) Schematic outlining the pipeline for identification of nucleophile sites and possible warhead incorporation sites. B) Model of minibinder designed to covalently target K108 in PD-L1 which was predicted by the ‘reactivity sphere’ script. C) Synthesis of malemide EBA probe for protein modification. D) Gel electrophoresis of designed PD-L1 minibinders incubated in a 1:10 molar equivalence with PD-L1. Covalent product formed at approximately 50 kDa. E) Surface plasmon resonance (SPR) binding analysis showing a reduction for non-covalent minibinder bound to PD-L1 following 10-minute dissociation, with almost no dissociation for MB1_EBA. F) Western blot analysis of covalent minibinder treated in A375 cells, a human melanoma cell line expressing PD-L1 (top) with Vinculin as a loading control (bottom) and specificity of designed PD-L1 minibinder in PD-L1 knock-out MDA-MB-231 TNBC cells assessed by flow cytometry. Fluorescence was only observed in cells expressing PD-L1, indicating the MB1_EBA is specific for PD-L1. G) Cell based assay confirms MB1_EBA potently blocks the native interaction between PD-L1 and PD-1. Data was collected in triplicate and data bars represent ± s.e.m.
