# Supplementary material for: Enhancing De Novo Designed Peptides and Proteins via Irreversible Covalent Isoquinolinium Capture
Source: ACS Chem Biol. Author manuscript; Available in PMC 2026 Oct 2. (PMC13630340; doi:10.1021/acschembio.6c00584)

Enhancing *De Novo* Designed Peptides and Proteins *via* Irreversible Covalent Isoquinolinium Capture

Paul M. Levine[1^#*], Patrick W. Erickson[1#], Timothy W. Craven[1^], Aaron T. Balana[^], Derrick R. Hicks[1$], Green Ahn[1], Chan J. Kim[1], Lisa Brandenburg[1@], Wei Yang[1&], Danielle P. Johnson Erickson[3], and David Baker[1,2*]

[1]Department of Biochemistry and Institute for Protein Design, University of Washington, Seattle, Washington 98195, United States

[2] HHMI, University of Washington, Seattle, Washington 98105

[3]Insamo, Berkeley, California 94704, United States

[3]Department of Radiation Oncology, School of Medicine, University of Washington, Seattle, WA 98195, USA

Current Address: [^]Insamo, Berkeley, California 94704, United States

Current Address: [&]Biobyte Solutions GmbH, Bothestr 142, 69126 Heidelberg, Germany

Current Address: [@]Department of Biosystems Science and Engineering, ETH Zürich, 4056 Basel, Switzerland

Corresponding Authors: paul@insamo.com [#]Authors Contributed Equally

Supplementary Information

Table of Contents:

Figure S1. Model of the MCB_D2:MCL1 complex. **Page S2**
Figure S2. Characterization of W1-W6. **Page S3**
Figure S3. Synthesis route for W4-W6. **Page S4**
Figure S4. Labeling at lower equivalence. **Page S4**
Figure S5. Site interrogation by blocking K234. **Page S5**
Figure S6. SPR binding via NHS of MCL1 with W3 or W6. **Page S5**
Figure S7. SPR binding via Biotin of MCL1 with W3 or W6. **Page S6**
Figure S8. Reactivity Sphere PD-L1 Output **Page S6**
Figure S9. Mass Spec Analysis of EBA Installation **Page S7**
Figure S10. SPR binding of PD-L1 with MB1 or MB1_EBA **Page S7**
Figure S11. Selectivity gel of minibinder with SIRT5 **Page S8**

Experimental Procedures **Page S9**

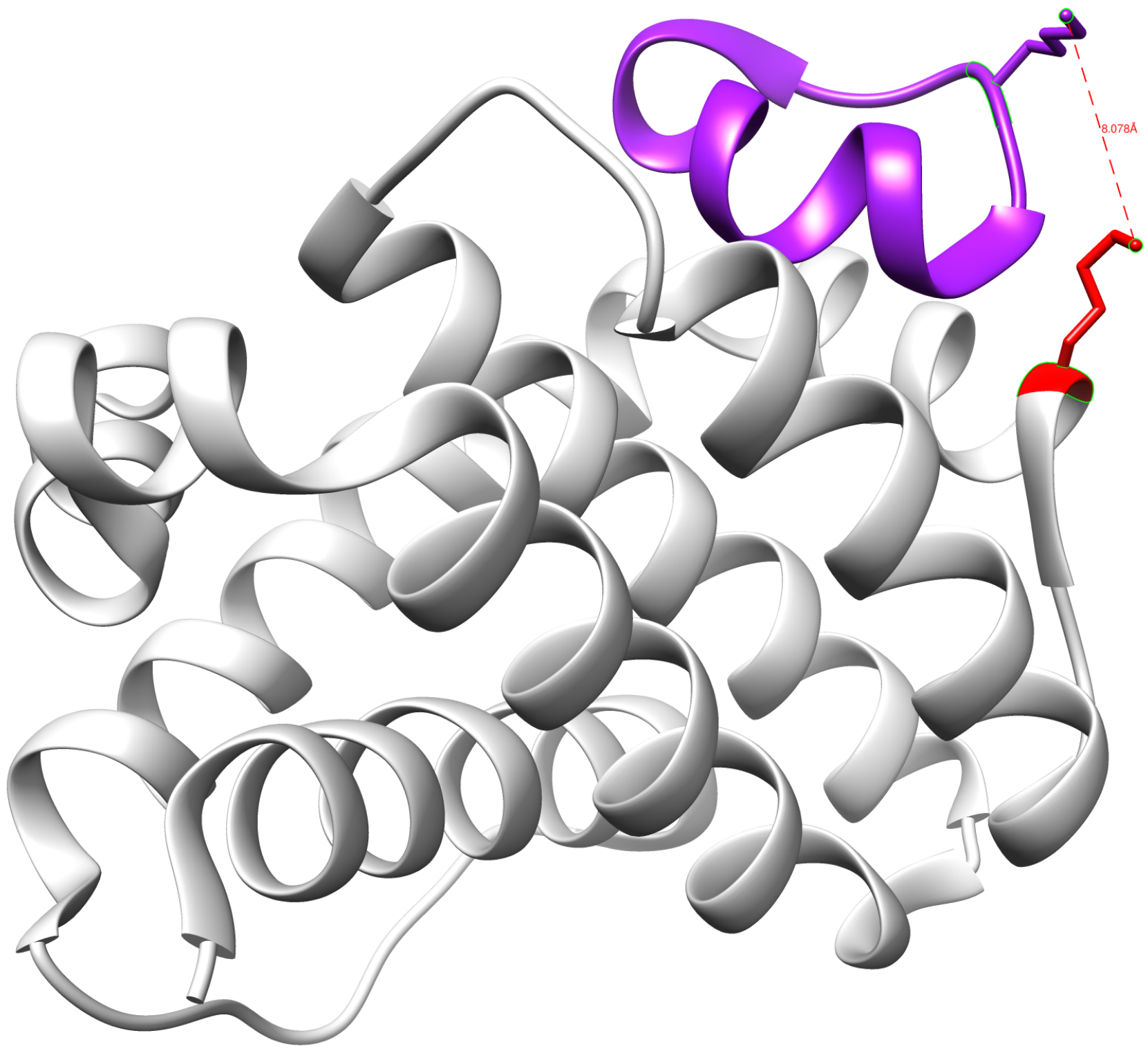

Figure S1. Model of the MCB_D2:MCL1 complex. Complex between MCL1 (gray) and MCB_D2 (purple) with K234 highlighted in red (PDB ID: 9CDT). The approximate distance between K234 and solvent exposed lysine in MCB_D2 is ~8.1Å.

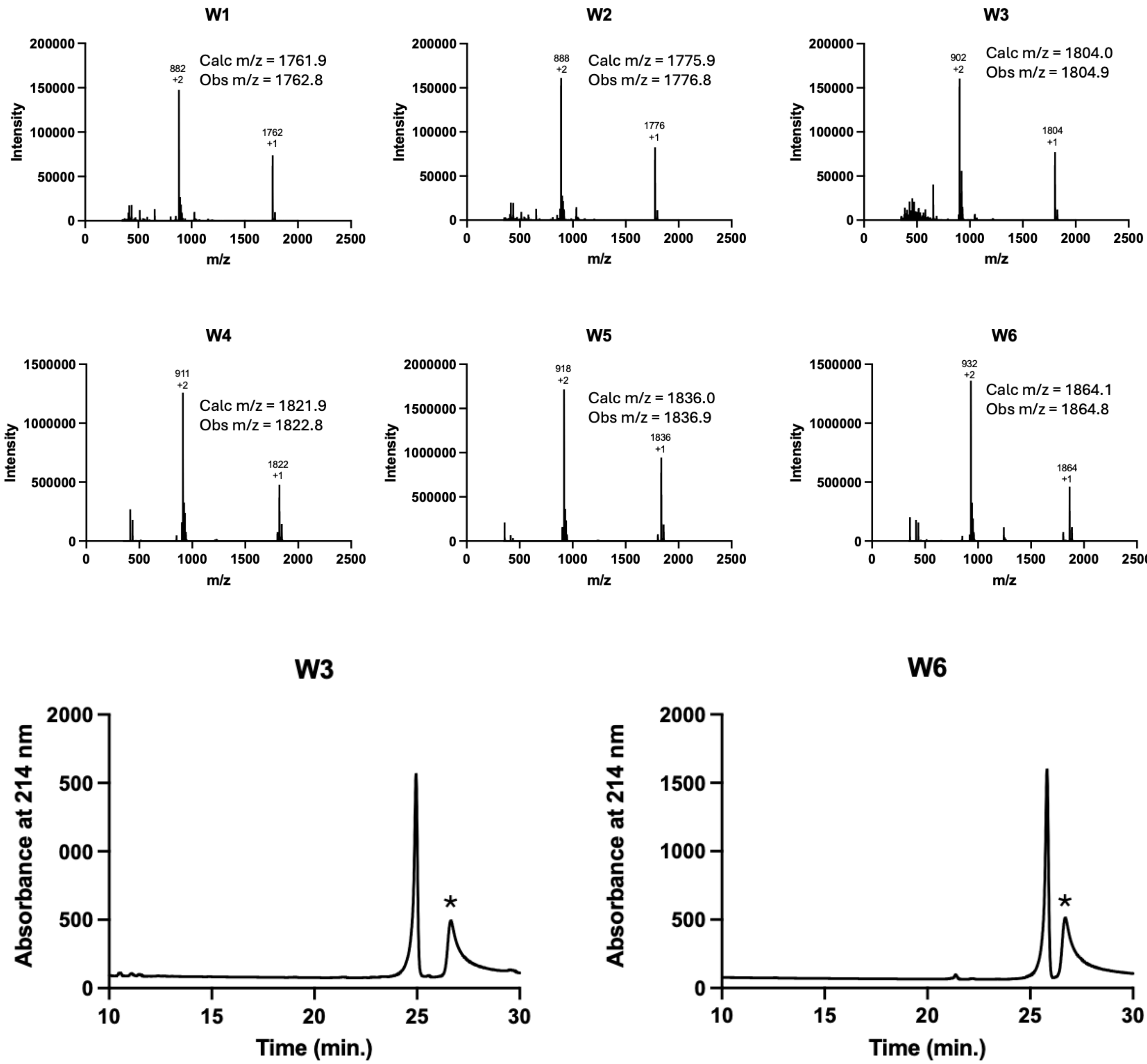

Figure S2. Characterization of W1-W6. Mass spectrometry and representative analytical RP-HPLC traces of the indicated synthetic peptides. Small peak at ~26.5 min is a column impurity.

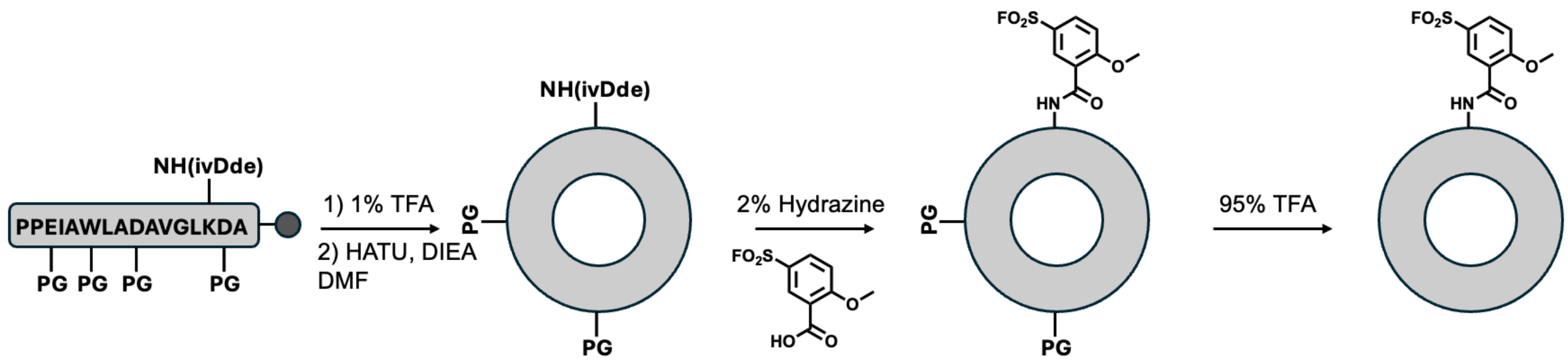

Figure S3. Synthesis route for W4-W6. Following linear synthesis, peptide was cleaved from resin in 1% TFA and cyclized under standard conditions (Boc and tBu protecting groups remain intact). Then, ivDde was selectively deprotected to install warhead prior to global cleavage.

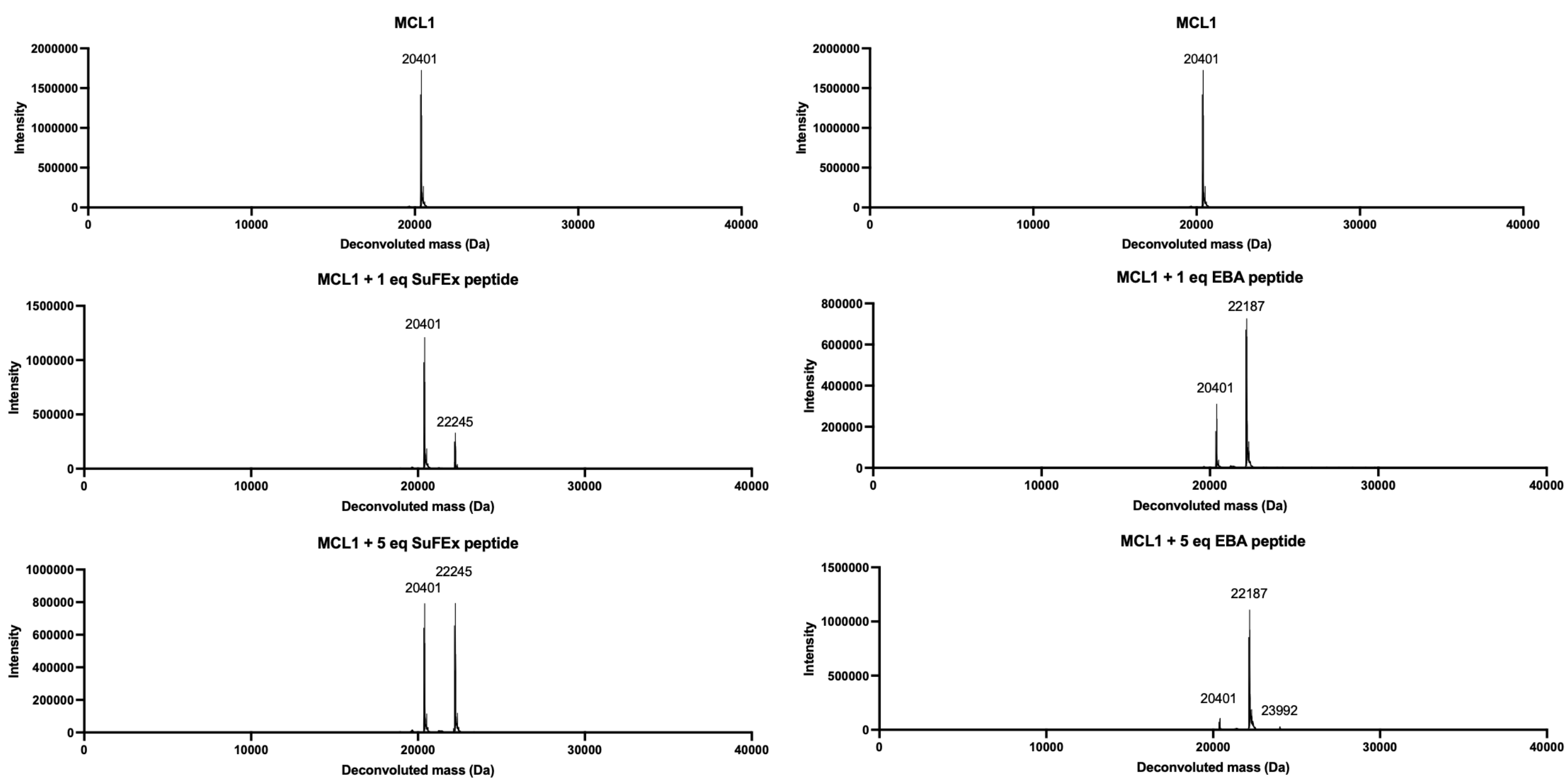

Figure S4. Peptides W3 and W6 irreversibly crosslink MCL1. Cross-linking is evaluated with either a 1:1 (2 μM) equivalence or 5-fold excess of the peptide (10 μM) over recombinant MCL-1 (a.a. residues D172-H320) (2 μM) at physiological pH and temperature for 2 h. Deconvoluted mass spectra show at 1 and 5 equivalence only single labeling with EBA is observed.

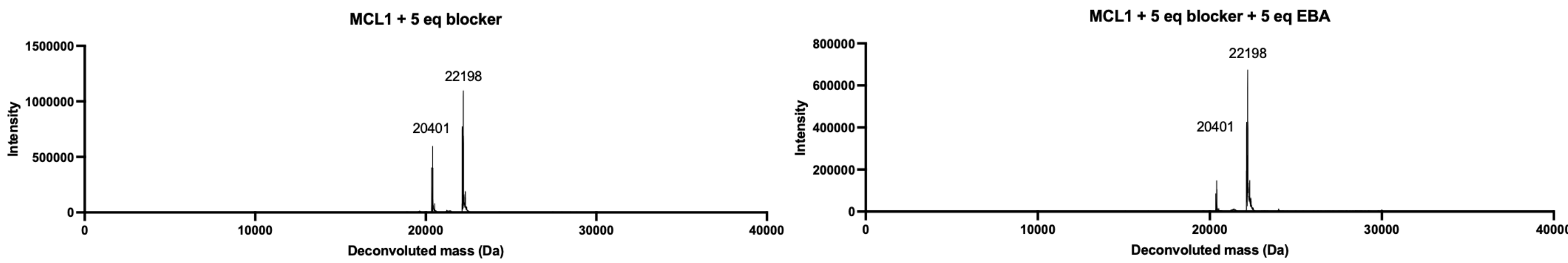

Figure S5. Site interrogation by blocking K234. Recombinant MCL-1 (a.a. residues D172-H320) (2 μM) was first reacted with a native substrate outfitted with a SuFEx known to covalently label K234. Following the reaction, the mixture was purified via 7k zeba column to remove any unreacted peptide and then 5 eq. of **W3** was added to see if additional labeling occurs elsewhere. No additional labeling was found, consistent with the target site being K234.

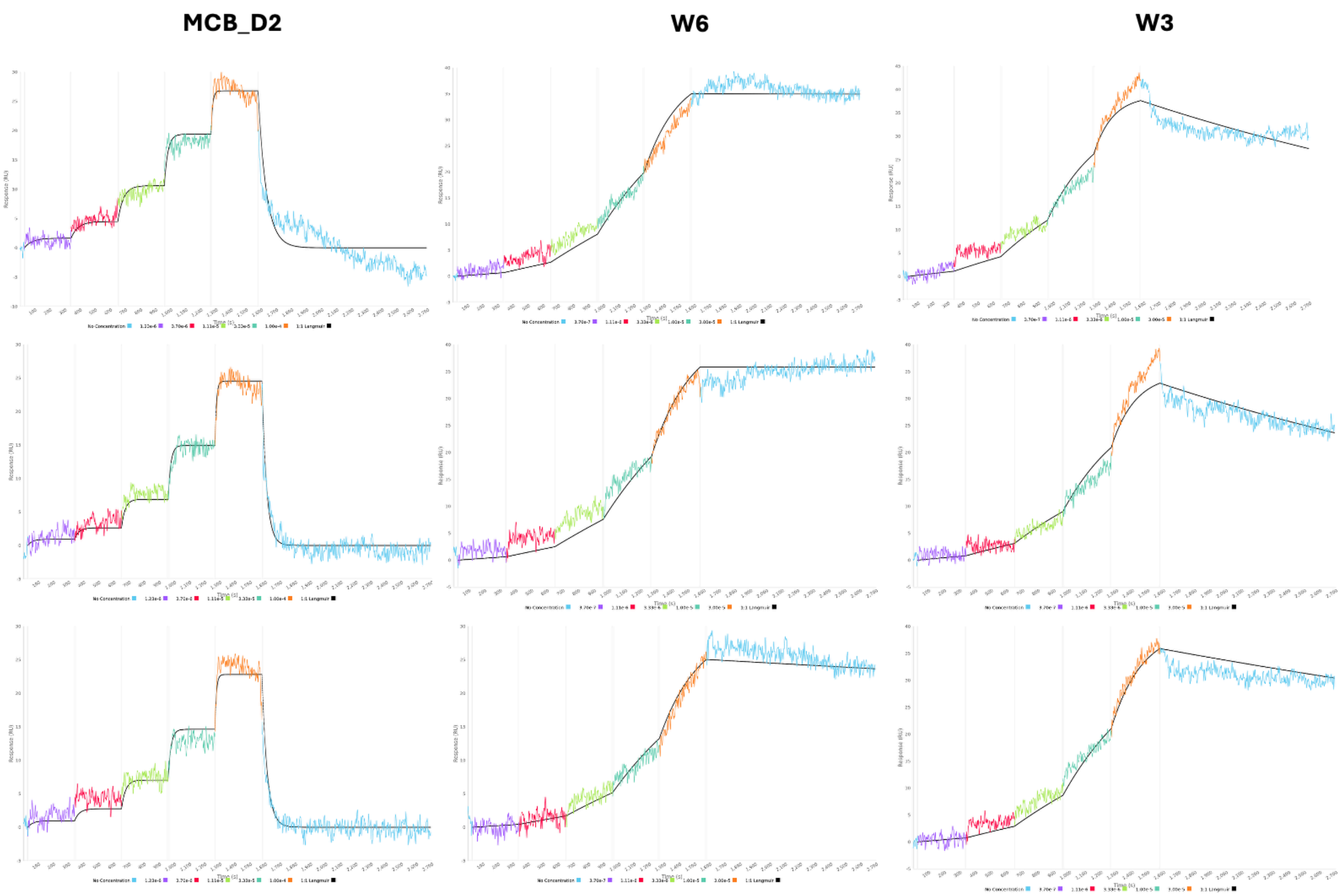

Figure S6. SPR analysis of MCL1 with peptides. SPR sensorgrams were conducted in triplicate.

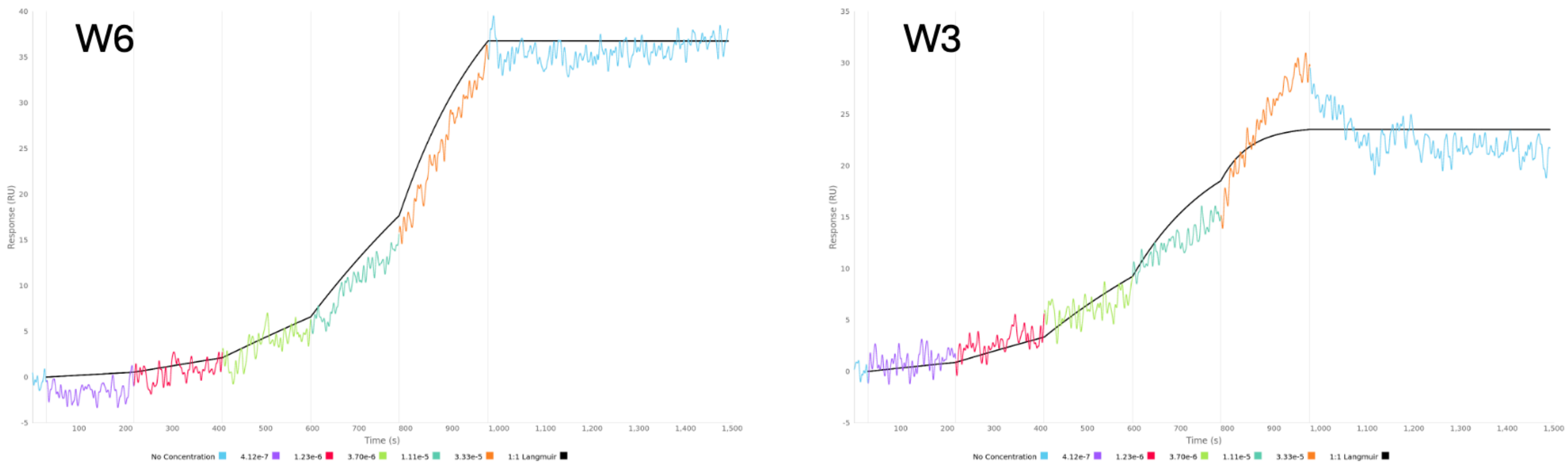

Figure S7. SPR sensorgrams of MCL1 with peptides via biotin capture.

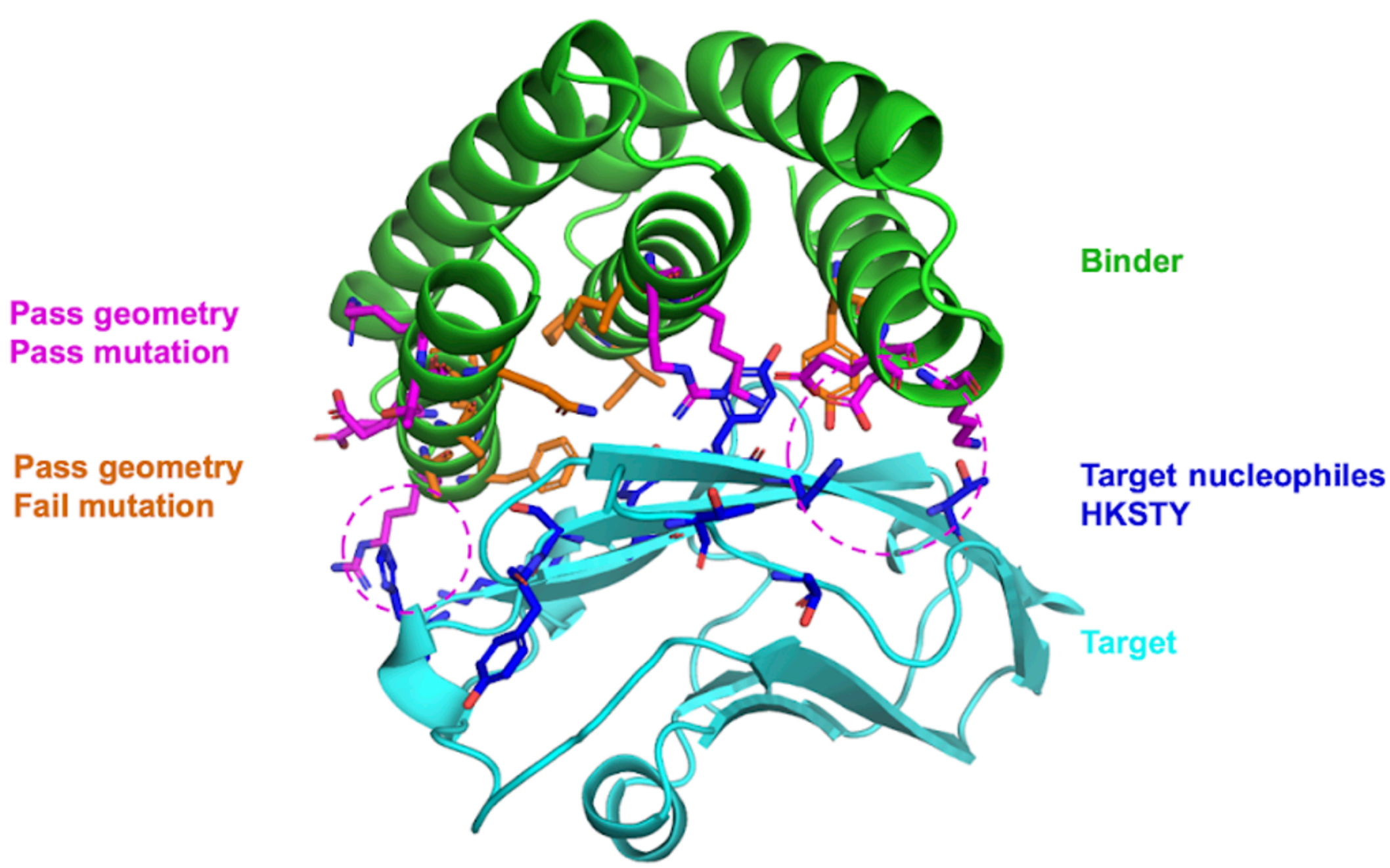

Figure S8. Representative example of our reactivity sphere script. Upon running the script, sites that pass mutation and geometry (purple) can be distinguished from sites that do not meet these requirements (orange).

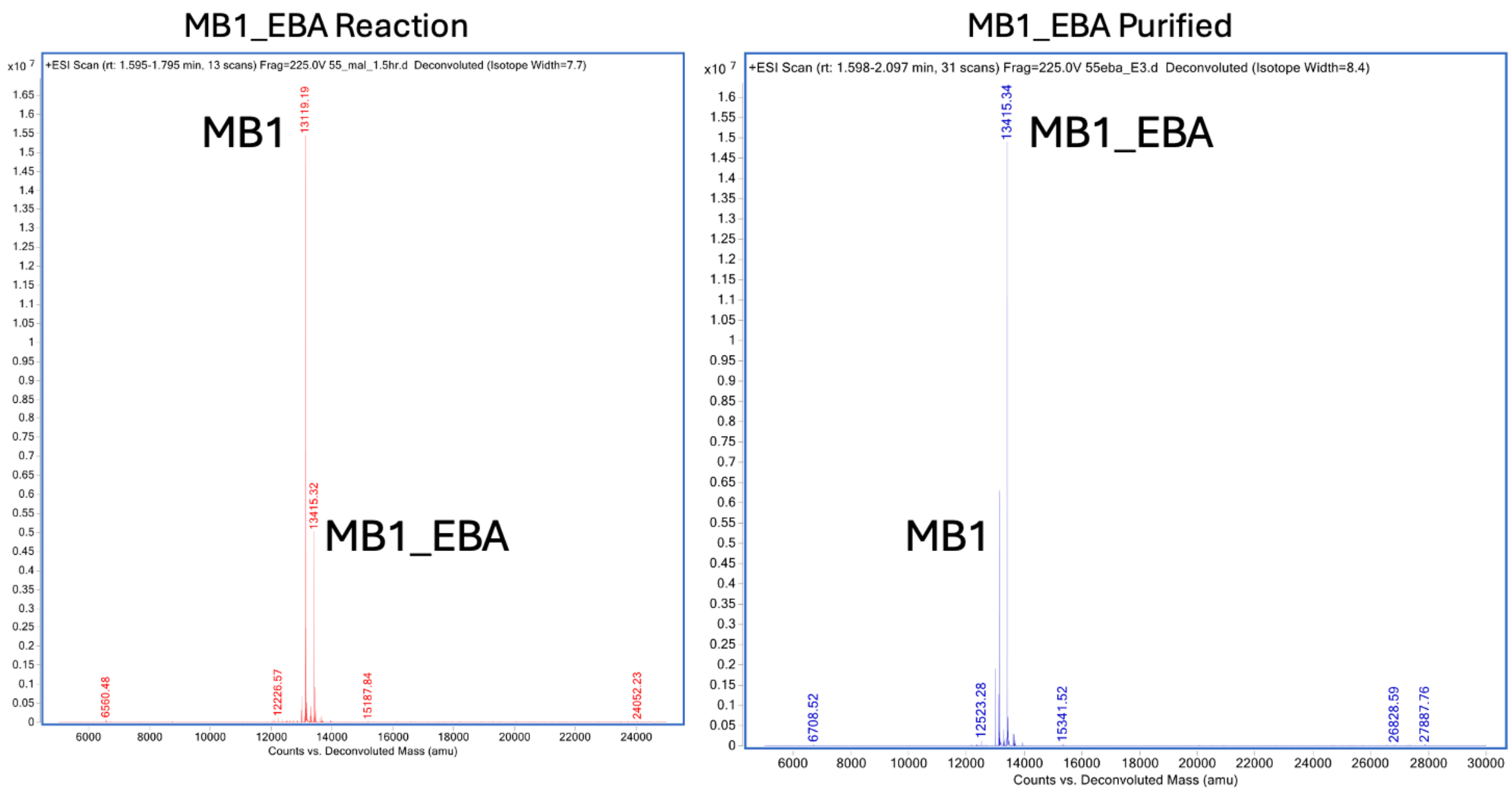

Figure S9. Mass spectrometry analysis of chemical installation of the EBA warhead onto MB1. The malemide probe was added in excess and only one modification on MB1 occurs, which can then be mostly purified from unlabeled protein.

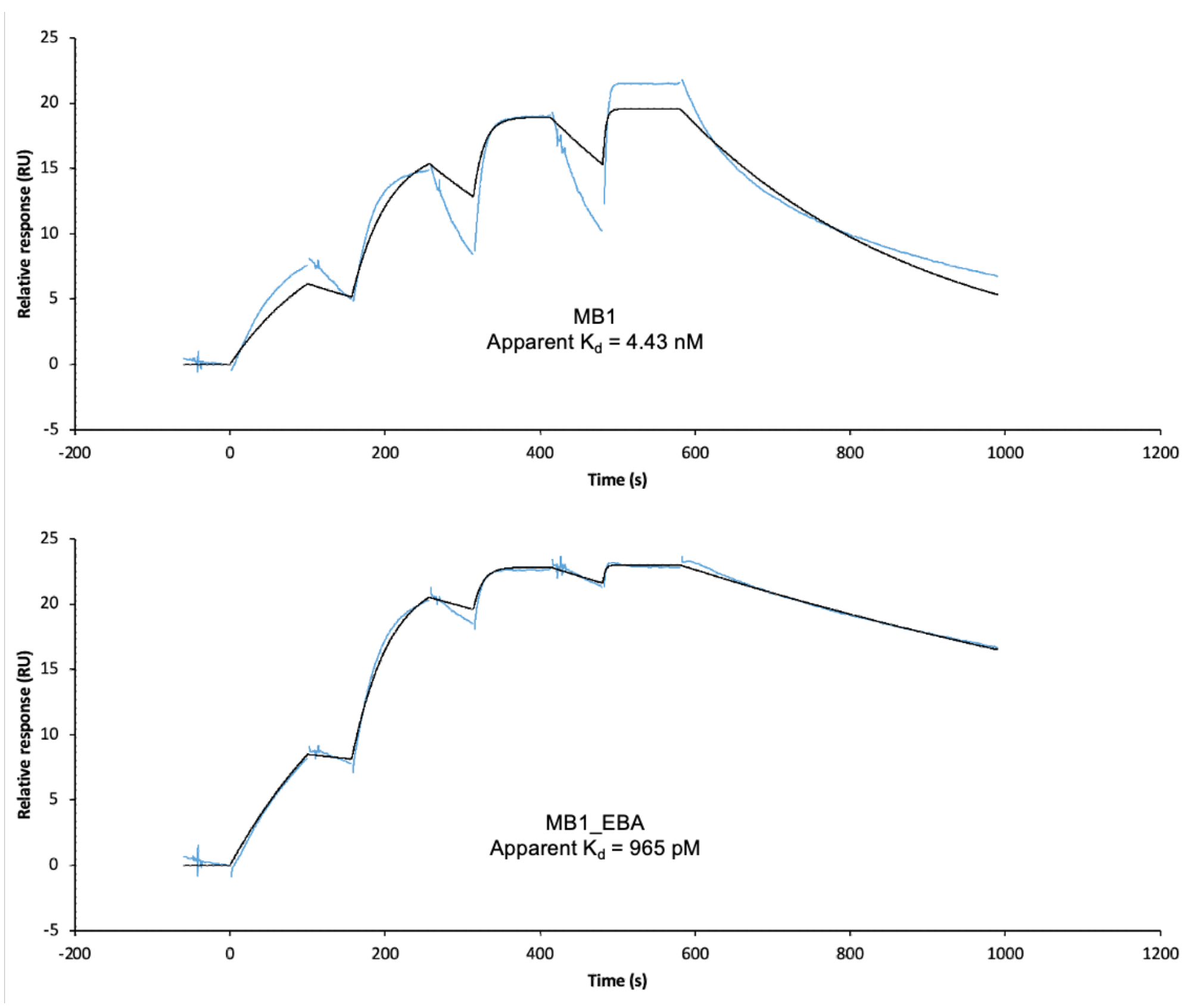

Figure S10. SPR sensograms for MB1 and MB1_EBA.

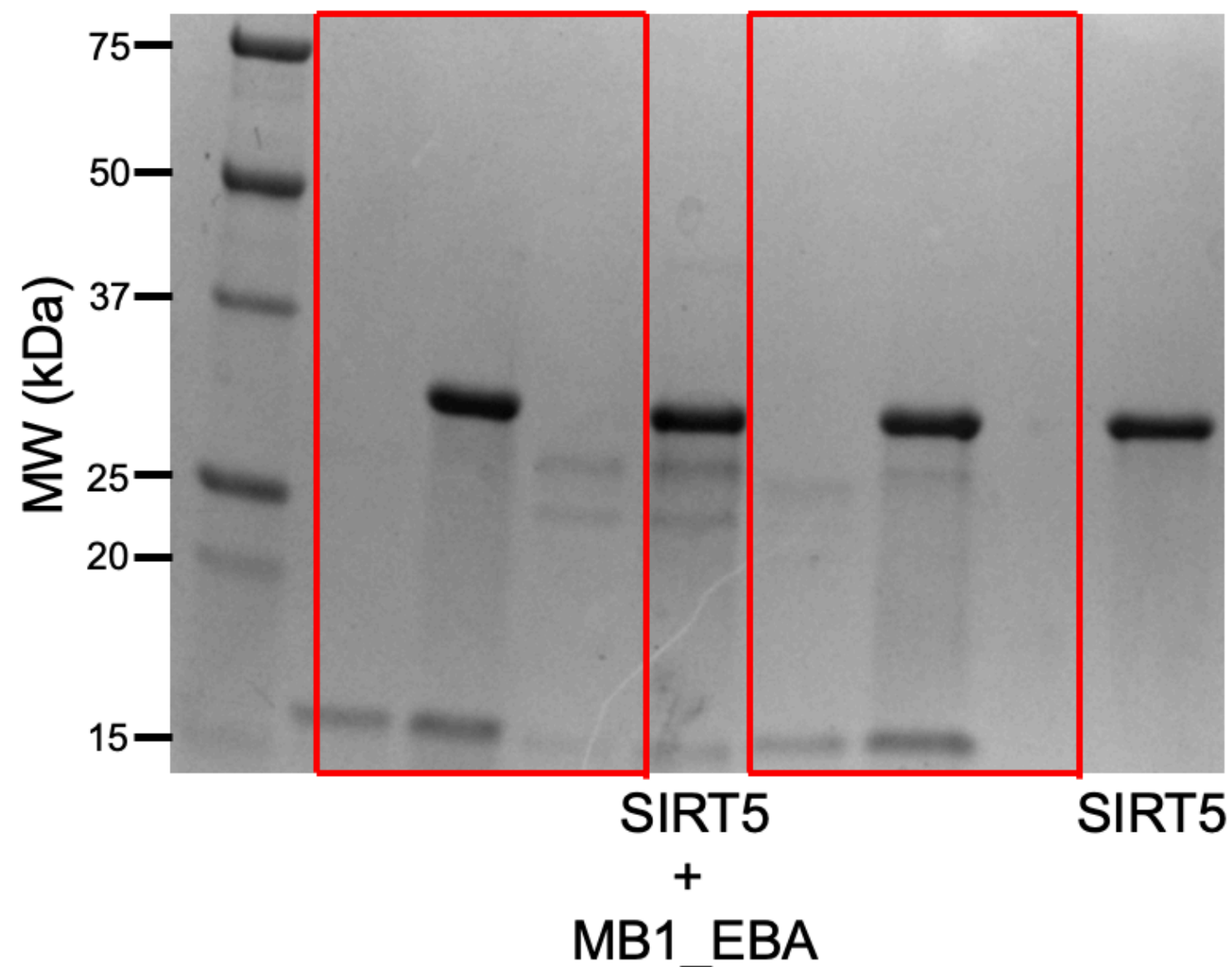

Figure S11. Selectivity assessment of MB1_EBA with a random protein SIRT5. In the presence of MB1_EBA, no higher molecular weight species were identified, indicating no reaction occurred between SIRT5 and MB1_EBA. This was conducted at 37°C for 16 hours (5uM SIRT5 + 5 uM MB1_EBA). The samples in the red boxes are not relevant to this manuscript.

EXPERIMENTAL PROCEDURES

***Materials*.**
MCL1-GST was purchased from Reaction Biology (Malvern, PA). PD-L1 was purchased from Acro. Biotinylated recombinant MCL-1 (a.a. residues D172-H320) was purchased from Sino Biologics. 5-(Fluorosulfonyl)-2-methoxybenzoic acid was acquired from AmBeed. All solvents and reagents were purchased from commercial sources (Sigma-Aldrich, Chem-Impex, etc.) and used without any further purification. All aqueous solutions were prepared using ultrapure laboratory grade water (deionized, filtered, and sterilized) obtained from an in-house ELGA water purification system. For analytical analysis, a C_{18} reversed-phase HPLC column was used (Higgins). Samples were eluted with a 5-95% acetonitrile/water gradient (0.1% TFA) in 45 minutes with a flow rate of 1 mL/min and monitored at 214 nm. For purifications, semi-preparative C_{18} reversed-phase HPLC columns were used (Higgins). Samples were eluted with a 25-95% acetonitrile/water gradient (0.1% TFA) in 35 minutes with a flow rate of 3.0 mL/min, and monitored at 214 nm. Mass spectra were acquired on an Agilent Technologies 1290 Series Q-TOF.

Peptide Synthesis.
All peptides were synthesized using standard Fmoc solid-phase chemistry on 2-Cl trityl (aapptec, 0.9 mmol/g) resin using PyAOP (5 equiv, Chem-Impex) and DIEA (10 equiv, Sigma) in DMF for 45 min. When linear SuFEx precursors were peptides were completed, they were selectively deprotected from resin (1:4:95 TFA/triisopropylsilane/dichloromethane) for 2 min. at room temperature 6 times and cyclized in DMF with HATU and DIEA. For SuFEx peptides, Lys(ivDde), Dap(ivDde), or Dab(ivDde) was then selectively deprotected with hydrazine (2% in DMF, 3x5 min.) for warhead installation. Crude peptides were then amide coupled to SuFEx warhead with DMF and HATU/DIEA and finally globally deprotected (95:2.5:2.5 TFA/water/triisopropylsilane for 3.5h) for final purification. For EBA peptides, linear peptides were generated and fully cleaved/globally deprotected from resin (95:2.5:2.5 TFA/water/triisopropylsilane for 3.5h) then head-to-tail cyclized using DMF with HATU/DIEA. For warhead installation, EBA probe **2** was added to purified peptide (4 eq.) in HEPES buffer (pH 8) for 2 hours. All peptides were subjected to preoperative HPLC and characterized by mass analysis and the and analytical HPLC.

Protein Expression.
Minibinder MB1 was prepared similarly according to our recent publication.[1]

Protein Labeling MCL1.
Peptides **W1-W6** (20uM, 10 equiv.) were added to GST-tagged MCL1 (2uM, 1.0 equiv.) in 50 mM HEPES, 500 mM NaCl, and 1 mM TCEP and incubated at 37 degrees for 2 hours. Following incubation, the reactions were analyzed by mass spectrometry. For labeling at lower equivalence, recombinant MCL-1 (a.a. residues D172-H320) was used.

Protein Labeling PDL1.
MB1_EBA (10uM, 10 equiv.) was added to PD-L1 (1uM, 1.0 equiv.) in 50 mM HEPES, 500 mM NaCl, and 1 mM TCEP and incubated at 37 degrees. At each time point, an aliquot of the reaction was removed, denatured, and frozen until all aliquots were collected. Following this, samples were loaded onto a gel and analyzed by gel electrophoresis.

Generation of PD-L1 KO Cells

MDA-MB-231 cells were cultured in Dulbecco's minimum essential medium (DMEM) supplemented with 10% heat-inactivated fetal bovine serum (HI-FBS) and 1% penicillin/streptomycin. To generate PD-L1 knockout (KO), wild-type (WT) MDA-MB-231 cells were nucleofected with a Lonza 4D-Nucleofector Unit using the Lonza SE Cell Line 4D-Nucleofector Kit. sgRNAs and spCas9 were obtained from Synthego's Gene Knockout Kit v2, and nucleofection was carried out using manufacturer's protocol.

Cell Surface Binding in WT and PD-L1 KO Cells

WT and KO cells were trypsinized and transferred to 96-U bottom plates. The cells were washed twice with ice-cold FACS buffer (0.02% BSA, DPBS) and incubated on ice with 100 nM of PD-L1 minibinder for 30 minutes. The cells were then washed once with FACS buffer, and stained with anti-His-antibody-488 on ice for 30 minutes. The cells were washed twice and analyzed on the Attune flow cytometer (Thermo Fisher).

Thermal Shift Assay.

MCL-1 melting curves were obtained using the Applied Biosystems Protein Thermal Shift kit (Thermo 4461146) using the standard manufacturer protocol. Recombinant MCL-1 (a.a. residues D172-H320) (stored in 20 mM HEPES 150 mM NaCl pH 7.4) at 2 uM final protein concentration was incubated with peptides at the indicated concentrations at a final volume of 20 uL. The final DMSO percentage in the melting reactions was 10%. Samples were chilled on ice for 30 minutes before loading onto a QuantStudio5, 96-well thermocycler. Samples were equilibrated at 25°C for 3 minutes, then heated to 99°C at a temperature ramp of 0.05°C/s. Raw thermal melting curves were normalized to the maximum fluorescence data point for each experiment.

SPR.

Binding of the peptides to MCL-1 was detected using a Nicoya Alto LSPR system. Recombinant MCL-1 (a.a. residues D172-H320) in 10 mM sodium acetate pH 6 buffer was directly immobilized onto the sensor surface at a protein concentration of 25 ug/mL using manufacturer's standard EDC/NHS coupling protocol. Binding experiments were performed in 20 mM HEPES, 150 mM NaCl, pH 7.4, 0.1% Tween 20, 3% DMSO. Five 3-fold dilutions of each peptides were flown over the protein, using 100 uM as the top concentration. The response at each concentration (at the end of a 300 second association time) was plotted against the concentrations of peptides used. Dissociation constants were obtained by fitting a nonlinear regression analysis using GraphPad Prism. Maximum peptide bound was determined using the final response measurement at 100 uM peptide concentration. The residual amount of bound peptide was also measured from the sensorgram after a 600s dissociation time.

Western Blot.

Cell pellets were stored at -80°C until lysis on ice with 1:10- cOmplete Ultra Protease Inhibitor: RIPA buffer for 30 minutes. Lysed samples were spun down at max speed for 10 mins at 4°C. Samples were then subjected to the Pierce 660nm Protein Assay to determine protein concentration and equilibrate samples to load 10ug/sample. Samples were run by SDS-PAGE on a 10% Criterion TGX Precast Midi Protein gel at 300 V for 30 minutes. The Chameleon Duo Pre-stained Protein ladder was used to verify protein size. Gels were transferred to nitrocellulose membrane using a Bio-RadTrans-blot Turbo. After transfer the membrane was blocked for 45 minutes at RT with Licor Blocking Buffer

prior to incubation with primary anti-PDL1 antibody (PD-L1 (E1L3N) XP Rabbit mAb) and/or anti-vinculin (Bio-rad MCA465GA mouse anti-human), diluted 1:1000, overnight at 4°C. Blots were then washed 3x with 1xTBST before incubation with the Licor IRDye 800CW Goat anti-Rabbit IgG and/or Goat anti-Mouse IgG secondary antibody at a dilution of 1:10,000 for 1 hour at RT. Blots were washed a second time with TBST and imaged using the Licor system.

HTRF Assay.
GST-tagged MCL-1 (4 nM) was incubated with FAM-Bak peptide (100 nM) in 20 mM potassium phosphate, pH 7.5, 50 mM NaCl, 1 mM EDTA, 0.005% Triton X-100, 1.0% DMSO. Following a 10 minute incubation, peptide was added *via* acoustic technology and then anti-GST-Tb was added. Peptides were allowed to compete for binding for 30 min. and then signal was measured. Curve fits were performed using GraphPad Prism software.

PD-L1 Assay.
Minibinder MB1 and MB1_EBA were tested in the PD-1/PD-L1 blockade bioassay (Promega) according to our recent publication.[2]

Reactivity Sphere Script.
Covalent crosslinking sites were identified using a PyRosetta-based structural workflow. A protein–protein complex structure was used to evaluate binder residues relative to target nucleophiles (Cys, Lys, His). Residue pairs were retained if the Cβ–Cβ distance (Cα for glycine) was <11.5 Å and side-chain vectors (Cα→Cβ) were oriented toward one another (dot product >0.3). Candidate binder residues were mutated *in silico* to alanine and lysine, followed by local side-chain repacking within 8 Å. Energetic effects were evaluated by computing binder monomer energy and interface ΔG using Rosetta. Residues were excluded if either mutation increased monomer or interface energy by >5 Rosetta Energy Units. Remaining residues were designated as candidate sites for covalent warhead incorporation.

```
import argparse
import csv
import json
import math
import os
import sys
from collections import defaultdict

import numpy as np

try:
    import pyrosetta
    from pyrosetta import rosetta
except ImportError as exc:
    raise SystemExit(
        "PyRosetta is required to run this script. Make sure PyRosetta is installed "
        "and available in your Python environment."
    ) from exc
```

```
def parse_args():
    parser = argparse.ArgumentParser(
        description="Prescreen binder residues for potential covalent warhead incorporation sites."
    )
    parser.add_argument("pdb", help="Input target/binder complex PDB file")
    parser.add_argument("--binder-chain", required=True, help="Binder chain ID (e.g. B)")
    parser.add_argument("--target-chain", required=True, help="Target chain ID (e.g. A)")
    parser.add_argument(
        "--nucleophiles",
        nargs="+",
        default=["CYS", "LYS", "HIS"],
        help="Target nucleophile residue types to consider (default: CYS LYS HIS)",
    )
    parser.add_argument(
        "--distance-cutoff",
        type=float,
        default=11.5,
        help="Maximum allowed Cb-Cb distance in Angstrom (default: 11.5)",
    )
    parser.add_argument(
        "--vector-dot-threshold",
        type=float,
        default=0.3,
         help="Minimum dot product for each Ca->Cb vector to point toward the partner residue (default:
0.3)",
    )
    parser.add_argument(
        "--energy-cutoff",
        type=float,
        default=5.0,
        help="Maximum allowed increase in monomer or binding energy in REU (default: 5.0)",
    )
    parser.add_argument(
        "--scorefxn",
        default="ref2015",
        help="Rosetta scorefunction name (default: ref2015)",
    )
    parser.add_argument(
        "--output-prefix",
        default="covalent_site_screen",
        help="Prefix for output files (default: covalent_site_screen)",
    )
    parser.add_argument(
        "--mute-rosetta",
        action="store_true",
        help="Mute most Rosetta output",
    )
    return parser.parse_args()
```

```
def init_pyrosetta(mute_rosetta=False):
    opts = []
    if mute_rosetta:
        opts.append("-mute all")
    pyrosetta.init(" ".join(opts))


def get_score_function(name):
    # Common case: ref2015 / beta_nov16 / etc.
    try:
        return pyrosetta.create_score_function(name)
    except Exception:
        # Fallback to default full-atom scorefunction.
        return pyrosetta.get_fa_scorefxn()


def get_chain_residues(pose, chain_id):
    residues = []
    for i in range(1, pose.total_residue() + 1):
        if pose.pdb_info().chain(i) == chain_id:
            residues.append(i)
    return residues


def pose_res_to_pdb_string(pose, resi):
    return pose.pdb_info().pose2pdb(resi).strip()


def xyz_to_np(v):
    return np.array([v.x, v.y, v.z], dtype=float)


def get_atom_xyz(residue, atom_name):
    if residue.has(atom_name):
        return xyz_to_np(residue.xyz(atom_name))
    return None


def get_cb_xyz(residue):
    cb = get_atom_xyz(residue, "CB")
    if cb is not None:
        return cb
    # Gly fallback
    return get_atom_xyz(residue, "CA")
```

```
def get_ca_xyz(residue):
    return get_atom_xyz(residue, "CA")


def safe_normalize(vec):
    norm = np.linalg.norm(vec)
    if norm < 1e-8:
        return None
    return vec / norm


def cb_cb_distance(res1, res2):
    cb1 = get_cb_xyz(res1)
    cb2 = get_cb_xyz(res2)
    if cb1 is None or cb2 is None:
        return None
    return float(np.linalg.norm(cb1 - cb2))


def sidechains_point_toward_each_other(res1, res2, threshold=0.3):
    ca1 = get_ca_xyz(res1)
    cb1 = get_cb_xyz(res1)
    ca2 = get_ca_xyz(res2)
    cb2 = get_cb_xyz(res2)

    if any(x is None for x in (ca1, cb1, ca2, cb2)):
        return False, None, None

    v1 = safe_normalize(cb1 - ca1)   # sidechain direction for residue 1
    v2 = safe_normalize(cb2 - ca2)   # sidechain direction for residue 2
    toward_2 = safe_normalize(cb2 - cb1)
    toward_1 = safe_normalize(cb1 - cb2)

    if any(x is None for x in (v1, v2, toward_2, toward_1)):
        return False, None, None

    dot1 = float(np.dot(v1, toward_2))
    dot2 = float(np.dot(v2, toward_1))

    return (dot1 > threshold and dot2 > threshold), dot1, dot2


def extract_chain_pose(pose, chain_id):
    try:
        chain_num = rosetta.core.pose.get_chain_id_from_chain(chain_id, pose)
        return pose.split_by_chain(chain_num)
    except Exception as exc:
        raise RuntimeError(
```

```
        f"Failed to extract chain '{chain_id}'. Check that the chain exists in the input PDB."
    ) from exc


def compute_total_energy(pose, scorefxn):
    return float(scorefxn(pose))


def compute_binder_monomer_energy(complex_pose, binder_chain, scorefxn):
    binder_pose = extract_chain_pose(complex_pose, binder_chain)
    return compute_total_energy(binder_pose, scorefxn)


def compute_binding_energy(complex_pose, target_chain, binder_chain, scorefxn):
    complex_energy = compute_total_energy(complex_pose, scorefxn)
    target_pose = extract_chain_pose(complex_pose, target_chain)
    binder_pose = extract_chain_pose(complex_pose, binder_chain)
    separated_energy = compute_total_energy(target_pose, scorefxn) +
compute_total_energy(binder_pose, scorefxn)
    return float(complex_energy - separated_energy)


def mutate_and_repack(pose, resi, new_res3, scorefxn):
    test_pose = pose.clone()

    mutator = rosetta.protocols.simple_moves.MutateResidue(resi, new_res3)
    mutator.apply(test_pose)

    task = rosetta.core.pack.task.standard_packer_task(test_pose)
    task.restrict_to_repacking()

    packer = rosetta.protocols.minimization_packing.PackRotamersMover(scorefxn, task)
    packer.apply(test_pose)

    return test_pose


def find_geometric_candidates(pose, binder_chain, target_chain, nucleophile_types, distance_cutoff,
vector_dot_threshold):
    binder_residues = get_chain_residues(pose, binder_chain)
    target_residues = get_chain_residues(pose, target_chain)

    candidates = []
    for b in binder_residues:
        b_res = pose.residue(b)
        for t in target_residues:
            t_res = pose.residue(t)
            if t_res.name3() not in nucleophile_types:
```

```
                continue

            dist = cb_cb_distance(b_res, t_res)
            if dist is None or dist >= distance_cutoff:
                continue

            vectors_ok, dot1, dot2 = sidechains_point_toward_each_other(
                b_res, t_res, threshold=vector_dot_threshold
            )
            if not vectors_ok:
                continue

            candidates.append(
                {
                    "binder_res": b,
                    "binder_name3": b_res.name3(),
                    "binder_pdb": pose_res_to_pdb_string(pose, b),
                    "target_res": t,
                    "target_name3": t_res.name3(),
                    "target_pdb": pose_res_to_pdb_string(pose, t),
                    "cb_cb_distance": dist,
                    "binder_dot": dot1,
                    "target_dot": dot2,
                }
            )

    return candidates


def filter_candidates_by_energy(pose, candidate_pairs, binder_chain, target_chain, scorefxn,
energy_cutoff):
    baseline_monomer = compute_binder_monomer_energy(pose, binder_chain, scorefxn)
    baseline_binding = compute_binding_energy(pose, target_chain, binder_chain, scorefxn)

    unique_binder_positions = sorted({c["binder_res"] for c in candidate_pairs})
    residue_results = {}

    for resi in unique_binder_positions:
        keep = True
        per_mutation = []

        for mut in ("ALA", "LYS"):
            mutated_pose = mutate_and_repack(pose, resi, mut, scorefxn)
            mut_monomer = compute_binder_monomer_energy(mutated_pose, binder_chain, scorefxn)
            mut_binding = compute_binding_energy(mutated_pose, target_chain, binder_chain, scorefxn)

            delta_monomer = float(mut_monomer - baseline_monomer)
            delta_binding = float(mut_binding - baseline_binding)
```

```
        per_mutation.append(
            {
                "mutation": mut,
                "delta_monomer_energy": delta_monomer,
                "delta_binding_energy": delta_binding,
            }
        )

        if delta_monomer > energy_cutoff or delta_binding > energy_cutoff:
            keep = False

    residue_results[resi] = {
        "keep": keep,
        "mutation_results": per_mutation,
    }

    filtered_pairs = []
    for c in candidate_pairs:
        resinfo = residue_results[c["binder_res"]]
        if resinfo["keep"]:
            row = dict(c)
            row["mutation_results"] = resinfo["mutation_results"]
            filtered_pairs.append(row)

    return filtered_pairs, residue_results, baseline_monomer, baseline_binding


def collapse_by_binder(filtered_pairs):
    by_binder = {}
    for c in filtered_pairs:
        b = c["binder_res"]
        if b not in by_binder:
            by_binder[b] = {
                "binder_res": b,
                "binder_name3": c["binder_name3"],
                "binder_pdb": c["binder_pdb"],
                "targets": [],
                "mutation_results": c["mutation_results"],
            }
        by_binder[b]["targets"].append(
            {
                "target_res": c["target_res"],
                "target_name3": c["target_name3"],
                "target_pdb": c["target_pdb"],
                "cb_cb_distance": c["cb_cb_distance"],
                "binder_dot": c["binder_dot"],
                "target_dot": c["target_dot"],
```

```
        }
    )
    return by_binder


def write_csv(output_csv, by_binder):
    with open(output_csv, "w", newline="") as handle:
        writer = csv.writer(handle)
        writer.writerow(
            [
                "binder_pose_res",
                "binder_pdb_res",
                "binder_name3",
                "target_pose_res",
                "target_pdb_res",
                "target_name3",
                "cb_cb_distance_A",
                "binder_vector_dot",
                "target_vector_dot",
                "delta_monomer_ALA",
                "delta_binding_ALA",
                "delta_monomer_LYS",
                "delta_binding_LYS",
            ]
        )

        for b in sorted(by_binder):
            info = by_binder[b]
            mut_map = {m["mutation"]: m for m in info["mutation_results"]}
            for t in info["targets"]:
                writer.writerow(
                    [
                        info["binder_res"],
                        info["binder_pdb"],
                        info["binder_name3"],
                        t["target_res"],
                        t["target_pdb"],
                        t["target_name3"],
                        f"{t['cb_cb_distance']:.3f}",
                        f"{t['binder_dot']:.3f}",
                        f"{t['target_dot']:.3f}",
                        f"{mut_map['ALA']['delta_monomer_energy']:.3f}",
                        f"{mut_map['ALA']['delta_binding_energy']:.3f}",
                        f"{mut_map['LYS']['delta_monomer_energy']:.3f}",
                        f"{mut_map['LYS']['delta_binding_energy']:.3f}",
                    ]
                )
```

```
def write_json(output_json, payload):
    with open(output_json, "w") as handle:
        json.dump(payload, handle, indent=2)


def main():
    args = parse_args()

    init_pyrosetta(mute_rosetta=args.mute_rosetta)
    scorefxn = get_score_function(args.scorefxn)

    pose = pyrosetta.pose_from_pdb(args.pdb)

    nucleophile_types = {x.upper() for x in args.nucleophiles}

    geometric_candidates = find_geometric_candidates(
        pose=pose,
        binder_chain=args.binder_chain,
        target_chain=args.target_chain,
        nucleophile_types=nucleophile_types,
        distance_cutoff=args.distance_cutoff,
        vector_dot_threshold=args.vector_dot_threshold,
    )

    filtered_pairs, residue_results, baseline_monomer, baseline_binding =
filter_candidates_by_energy(
        pose=pose,
        candidate_pairs=geometric_candidates,
        binder_chain=args.binder_chain,
        target_chain=args.target_chain,
        scorefxn=scorefxn,
        energy_cutoff=args.energy_cutoff,
    )

    by_binder = collapse_by_binder(filtered_pairs)

    output_csv = f"{args.output_prefix}.csv"
    output_json = f"{args.output_prefix}.json"

    write_csv(output_csv, by_binder)
    write_json(
        output_json,
        {
            "input_pdb": args.pdb,
            "binder_chain": args.binder_chain,
            "target_chain": args.target_chain,
            "nucleophiles": sorted(nucleophile_types),
```

```
            "distance_cutoff": args.distance_cutoff,
            "vector_dot_threshold": args.vector_dot_threshold,
            "energy_cutoff": args.energy_cutoff,
            "baseline_binder_monomer_energy": baseline_monomer,
            "baseline_binding_energy": baseline_binding,
            "n_geometric_candidate_pairs": len(geometric_candidates),
            "n_final_candidate_pairs": len(filtered_pairs),
            "n_final_candidate_binder_residues": len(by_binder),
            "candidates": by_binder,
        },
    )

    print(f"Input PDB: {args.pdb}")
    print(f"Binder chain: {args.binder_chain}")
    print(f"Target chain: {args.target_chain}")
    print(f"Target nucleophile types: {', '.join(sorted(nucleophile_types))}")
    print(f"Geometric candidate pairs: {len(geometric_candidates)}")
    print(f"Final candidate pairs after energy filtering: {len(filtered_pairs)}")
    print(f"Final candidate binder residues: {len(by_binder)}")
    print(f"CSV output: {os.path.abspath(output_csv)}")
    print(f"JSON output: {os.path.abspath(output_json)}")

    if by_binder:
        print("\nPotential warhead-incorporation sites:")
        for b in sorted(by_binder):
            info = by_binder[b]
            print(f"\nBinder residue {info['binder_res']} ({info['binder_name3']} {info['binder_pdb']})")
            print("  Nearby target nucleophiles:")
            for t in info["targets"]:
                print(
                    f"    - {t['target_name3']} {t['target_pdb']} "
                    f"(pose {t['target_res']}), "
                    f"Cb-Cb={t['cb_cb_distance']:.2f} A, "
                    f"binder_dot={t['binder_dot']:.2f}, target_dot={t['target_dot']:.2f}"
                )
            print("  Mutation tolerance:")
            for m in info["mutation_results"]:
                print(
                    f"    - {m['mutation']}: dMonomer={m['delta_monomer_energy']:.2f}, "
                    f"dBinding={m['delta_binding_energy']:.2f}"
                )
    else:
        print("\nNo binder residues survived all filters.")


if __name__ == "__main__":
    main()
```

References

1. Inoue, N.; Onoda, A.; Hayashi, T. Site-Specific Modification of Proteins Through N-Terminal Azide Labeling and a Chelation-Assisted CuAAC Reaction. Bioconjugate Chem. **2019**, 30, 9, 2427–2434.
2. Yang, W.; Hicks, D. R.; Ghosh, A.; Schwartze, T A.; Coventry, B.; Goreshnik, I.; Allen, A.; Halabiya, S. F.; Chan, J. K.; Hinck, C. S.; Lee, D. S.; Bera, A. K.; Li, Z.; Wang, Y.; Schlichthaerle. T.; Cao. L.; Huang, B.; Garrett, S.; Gerben, S. R.; Rettie, S.; Heine, P, Murray, A.; Edman, N.; Carter, L.; Stewart, L.; Almo, S. C.; Hinck, A. P.; Baker, D. Design of High-Affinity Binder to Immune Modulating Receptors for Cancer Immunotherapy. Nat. Commun. **2025**, 16, 2001.

Synthesis of Targets 1-3.

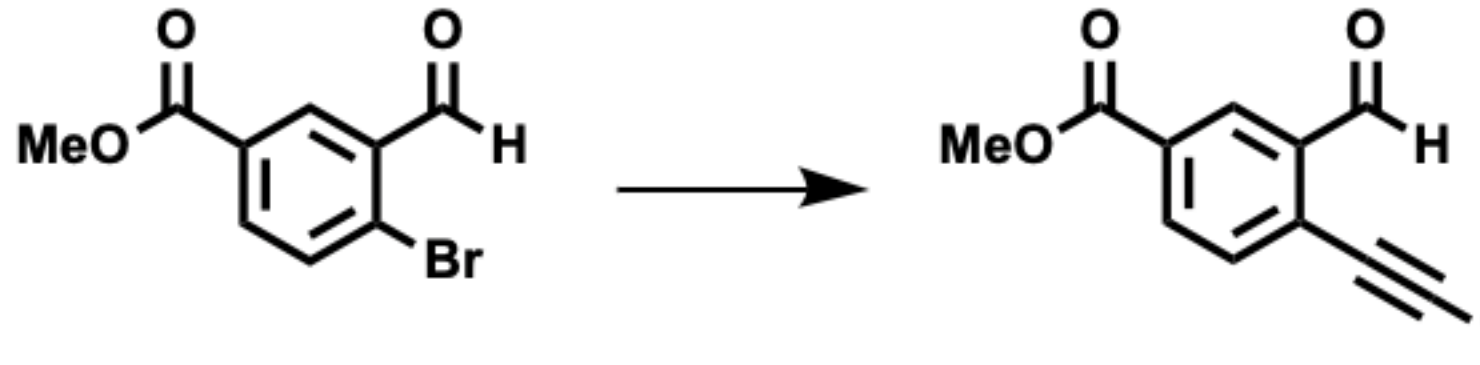

(C2)

A mixture of methyl 4-bromo-3-formyl-benzoate (5.00 g, 20.6 mmol), ethynyl(trimethyl)silane (2.42 g, 24.7 mmol), $Pd(PPh_3)_2Cl_2$ (722 mg, 1.03 mmol), CuI (118 mg, 617 µmol) and DIEA (6.65 g, 51.4 mmol) in THF (50 mL) was degassed and purged with N_2 3 times. The mixture was then stirred at 50°C for 2 h under N_2 atmosphere. The mixture was concentrated under vacuum to give a residue that was purified by flash silica gel chromatography (ISCO®; 40 g SepaFlash® Silica Flash Column, eluent of 0~10% petroleum ether/ethyl acetate with a gradient of 25 mL/min) to give compound **2** (3.00 g, 53.3% yield, 95.14% purity) as a colorless oil. ^{1}H NMR**:** *δ* 10.56 (s, 1H), 8.56 (d, *J* = 1.8 Hz, 1H), 8.20 - 8.18 (m, 1H), 7.65 (d, *J* = 8.0 Hz, 1H), 3.96 (s, 3H), 0.30 (s, 9H). $CDCl_3$

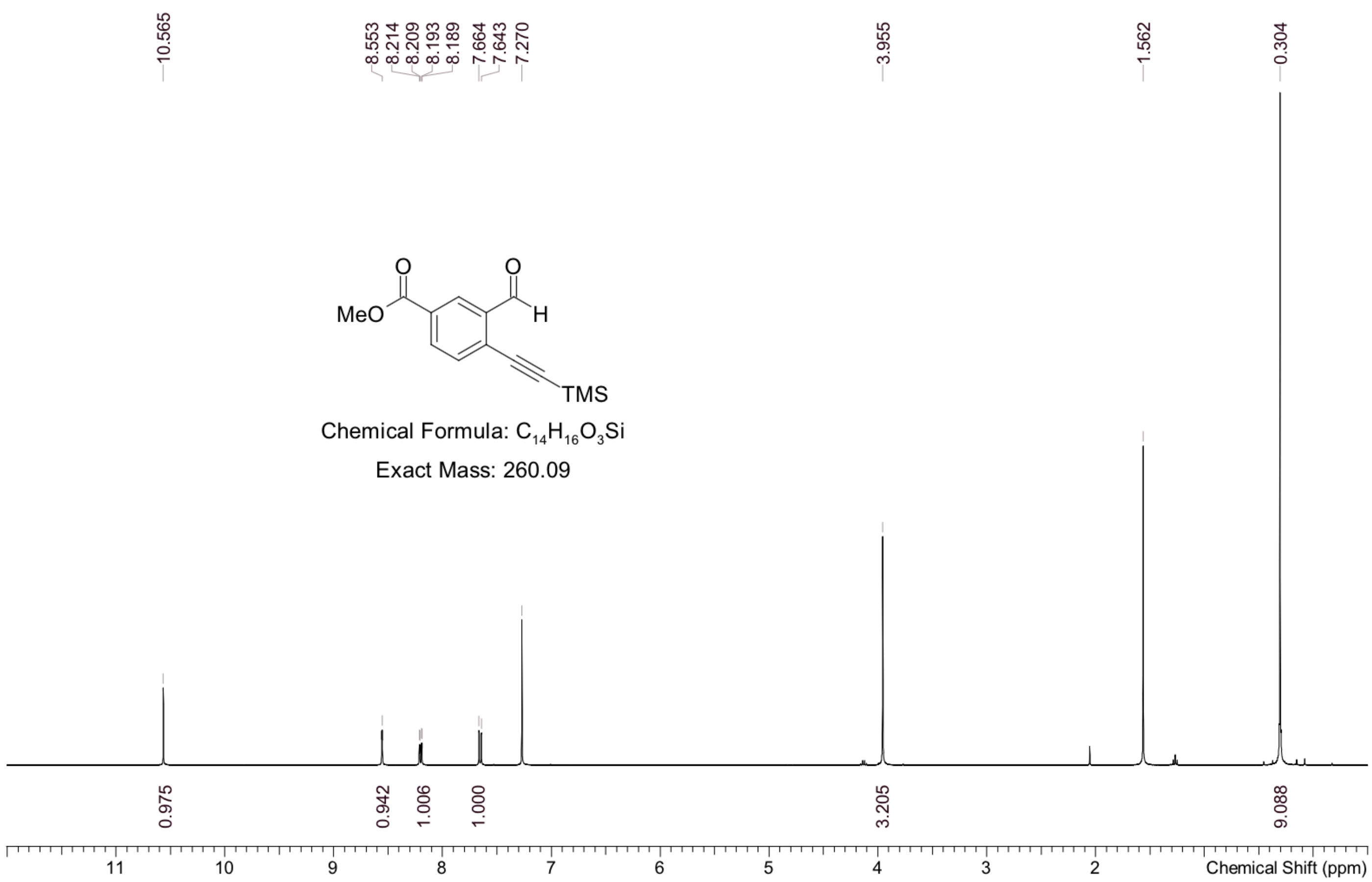

^{1}H NMR (400 MHz, $CDCl_3$) δ 10.56 (s, 1H), 8.56 (d, *J* = 1.8 Hz, 1H), 8.20 - 8.18 (m, 1H), 7.65 (d, *J* = 8.0 Hz, 1H), 3.96 (s, 3H), 0.30 (s, 9H)

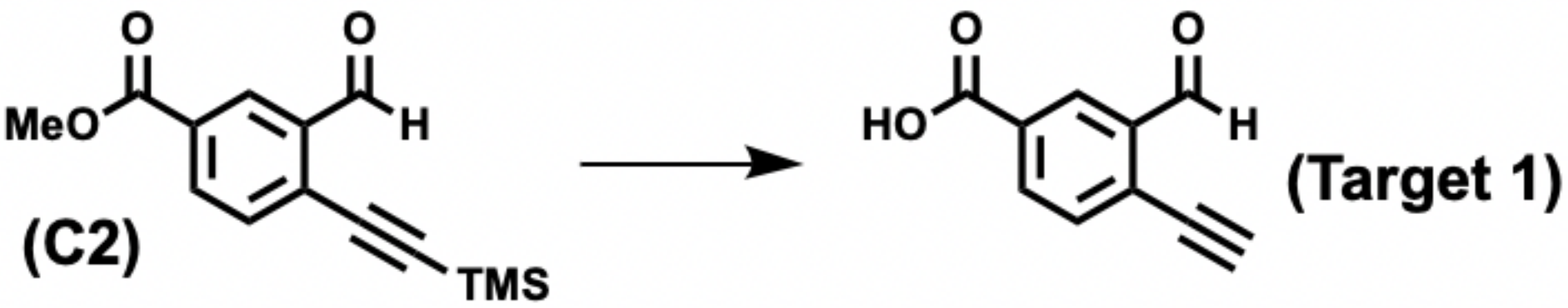

To a solution of methyl 3-formyl-4-(2-trimethylsilylethynyl)benzoate (2.50 g, 9.60 mmol) in THF (20 mL) was added LiOH·H_2O (604 mg, 14.4 mmol) and H_2O (5 mL) at 0 °C. The mixture was stirred at 25°C for 2 h and then poured into H_2O (20 mL) and extracted with EtOAc (30 mL). The aqueous layer was then adjusted pH to 4~5 with 1N HCl and extracted with EtOAc (30 mL x 2). The combined organic layers were washed with sat. brine (50 mL), dried over anhydrous Na_2SO_4 and concentrated *in vacuo* to give a residue that was purified by flash silica gel chromatography (ISCO®; 20 g SepaFlash® Silica Flash Column, eluent of 0~30% petroleum ether/ethyl acetate with a gradient of 16 mL/min) to give compound target 1 (293.8 mg, 1.64 mmol, 17.10% yield, 97.33% purity). ^{1}H NMR**:** *δ* 13.63 - 13.36 (m, 1H), 10.39 (s, 1H), 8.34 (d, *J* = 1.8 Hz, 1H), 8.19 (m, 1H), 7.80 (d, *J* = 8.0 Hz, 1H), 4.94 (s, 1H). DMSO-d_6

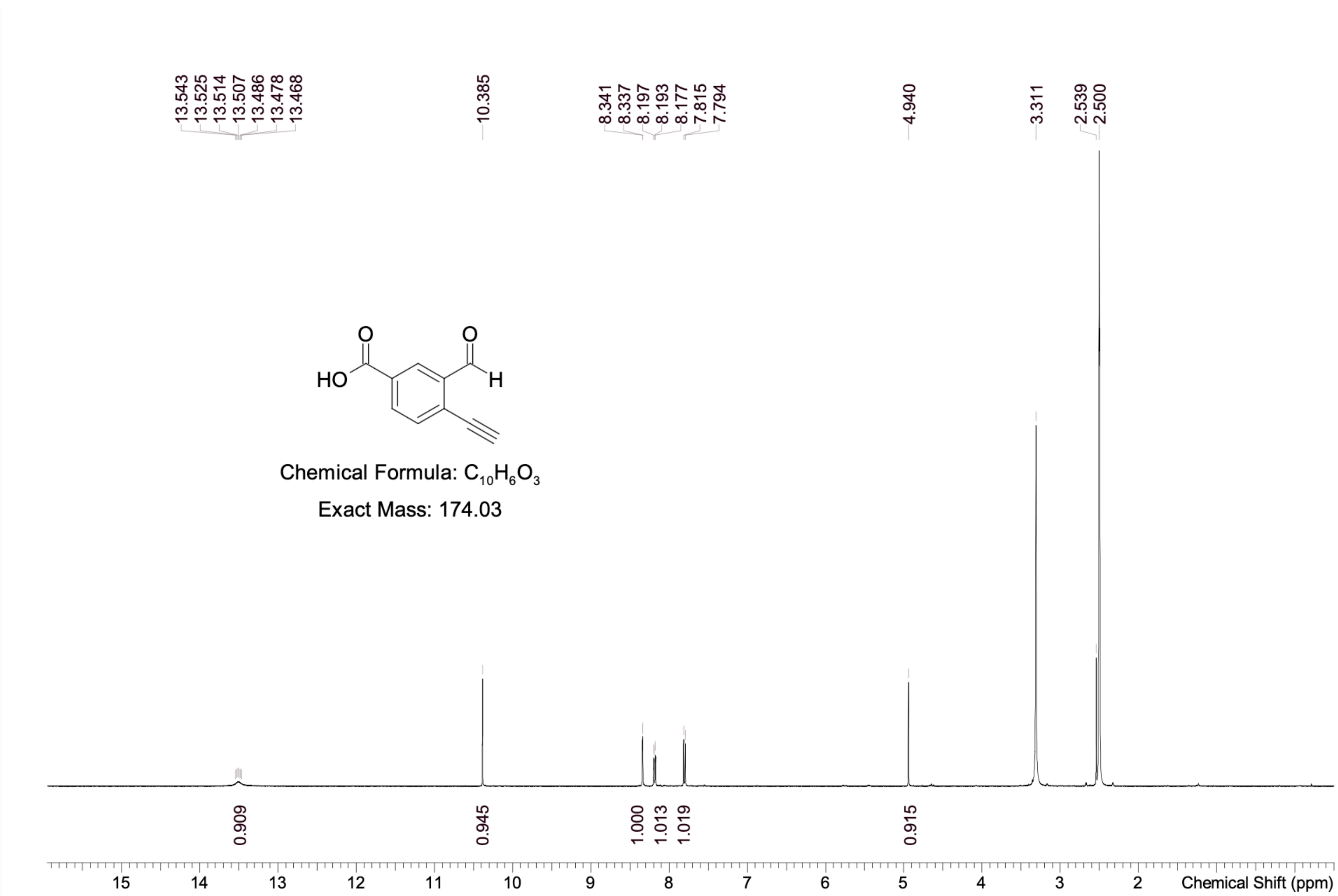

^{1}H NMR (400 MHz, DMSO-d_6) δ 13.63 - 13.36 (m, 1H), 10.39 (s, 1H), 8.34 (d, *J* = 1.8 Hz, 1H), 8.19 (m, 1H), 7.80 (d, *J* = 8.0 Hz, 1H), 4.94 (s, 1H)

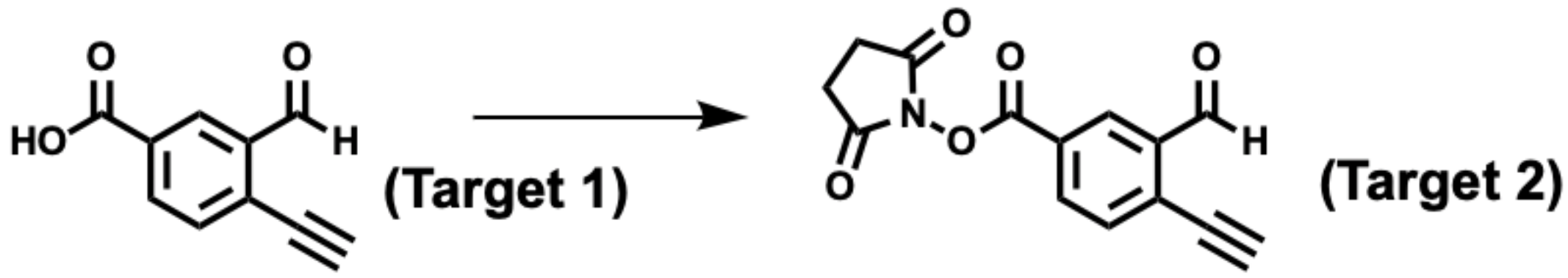

To a solution of 4-ethynyl-3-formyl-benzoic acid (400 mg, 1.61 mmol) in DMF (8 mL) was added DIPEA (312 mg, 2.41 mmol) and HATU (734 mg, 1.93 mmol) and the mixture was stirred at 25°C for 0.5 h. Then 1-hydroxypyrrolidine-2,5-dione (194 mg, 1.69 mmol) was added and the mixture was stirred at 25 °C for 2 h. The mixture was then diluted with H_2O (10 mL) and extracted with EtOAc (3 × 10 mL). The combined organic layers were washed with sat. brine (20 mL), dried over anhydrous Na_2SO_4 and concentrated *in vacuo* to give a residue that was purified by flash silica gel chromatography (ISCO®; 20 g SepaFlash® Silica Flash Column, eluent of 0~30% petroleum ether/ethyl acetate with a gradient of 16 mL/min) to yield target 2 (150 mg, 29.81% yield, 86.65% purity) was as a white solid. ^{1}H NMR**:** δ 10.38 (s, 1H), 8.44 (s, 1H), 8.34 (d, J = 7.2 Hz, 1H), 7.94 (d, J = 8.0 Hz, 1H), 5.12 (s, 1H), 2.91 (br s, 4H). DMSO-d_6

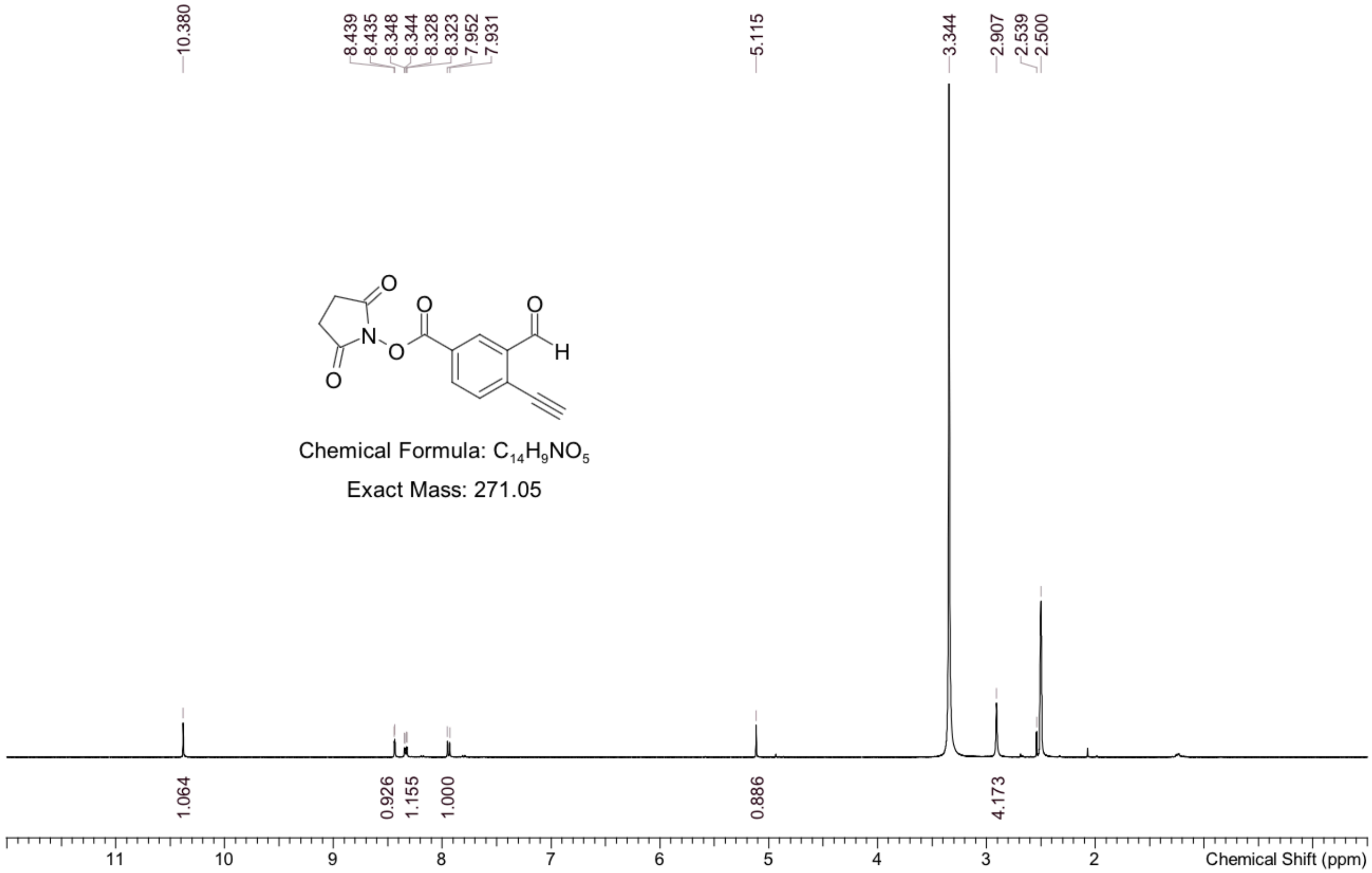

^{1}H NMR (400 MHz, DMSO-d_6) δ 10.38 (s, 1H), 8.44 (d, J = 1.6 Hz, 1H), 8.34 - 8.32 (m, 1H), 7.94 (d, J = 8.0 Hz, 1H), 5.12 (s, 1H), 2.91 (s, 4H)

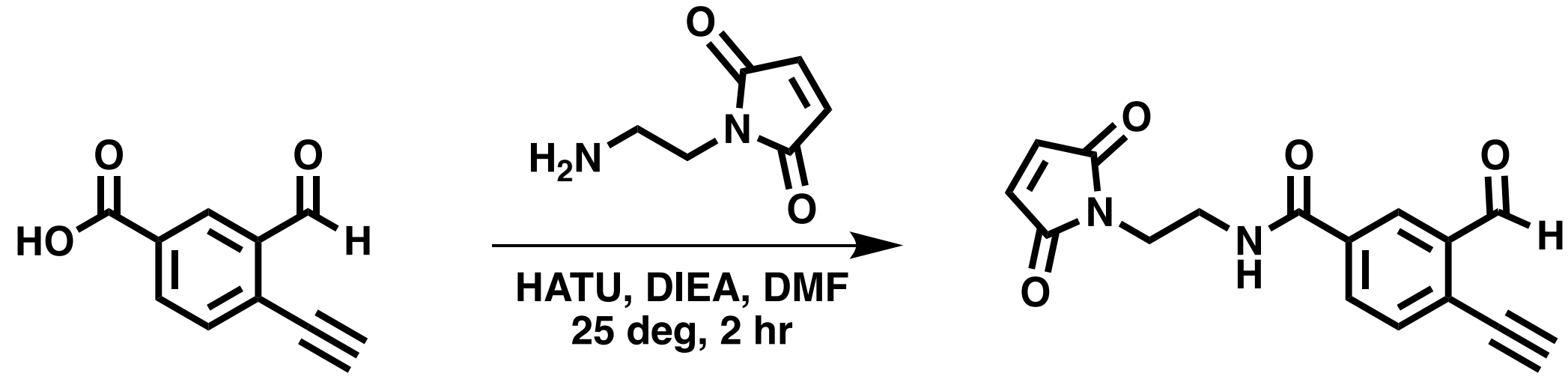

Target 3 (right): 2-ethynyl-5-carboxybenzaldehyde (1.50 g, 8.61 mmol, 1.00 eq) was added into a 250 mL round-bottom. To this, DMF (15.0 mL), DIEA (1.67 g, 12.9 mmol, 2.25 mL, 1.50 eq), and HATU (3.93 g, 10.3 mmol, 1.20 eq) were added and the mixture was stirred at 25°C for 30 minutes. Following this, 1-(2-aminoethyl)maleimide (1.60 g, 9.04 mmol, 1.05 eq) was added and the reaction continued to stir for 90 minutes. Upon consumption of 2-ethynyl-5-carboxybenzaldehyde, which was monitored by mass spec, the mixture was poured into H_2O (200 mL) and extracted with ethyl acetate (200 mL x 3). The combined organic layer was washed with brine (200 mL x 3) and then dried over Na_2SO_4, filtered, and concentrated under vacuum. The crude product was then purified by prep-HPLC (mobile phase: [water (HCl)-ACN]; gradient: 7%-37% B over 25 min) to give Mal_EBA (491.49 mg, 1.65 mmol, 19.2% yield, 99.6% purity) as a yellow solid, which confirmed by ^{1}H NMR and mass spectrometry. δ 10.39 (s, 1H), 8.90 - 8.87 (m, 1H), 8.22 (d, J = 2.00 Hz, 1H), 8.04 - 8.02 (m, 1H), 7.78 (d, J = 8.00 Hz, 1H), 7.01 (s, 2H), 4.88 (s, 1H), 3.60 - 3.58 (m, 2H), 3.44 - 3.40 (m, 2H). DMSO-d_6

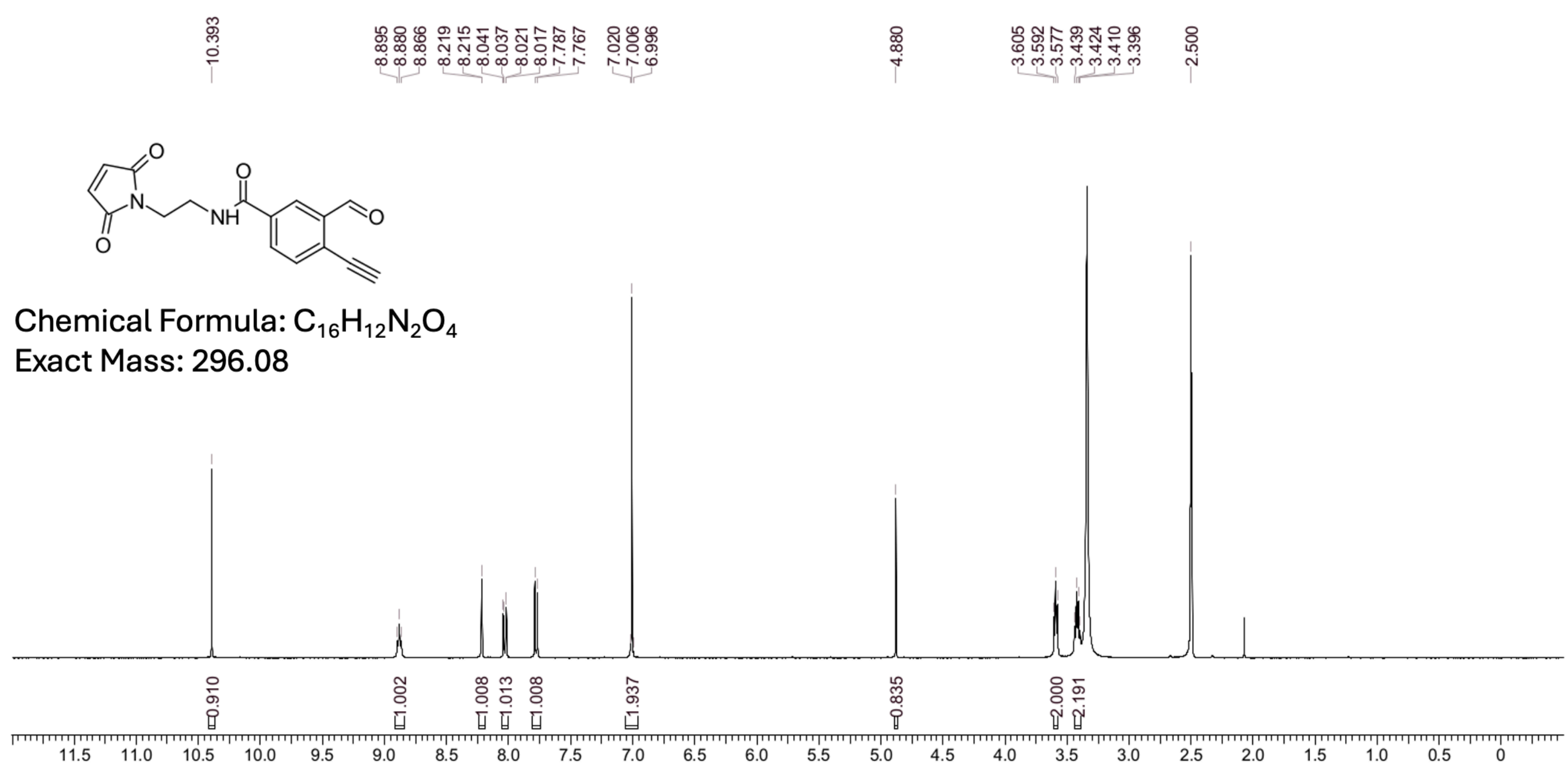

Supplement: Supplementary file 1 [file NIHMS2209922-supplement-Supplementary_Material.pdf]
